# Susceptibility of mice to primary *Echinostoma caproni* infections is associated with metabolic and structural changes

**DOI:** 10.3389/fimmu.2026.1720183

**Published:** 2026-04-21

**Authors:** Emma Fiallos, Paola Cociancic, José Guillermo Esteban, Carla Muñoz-Antoli, Rafael Toledo

**Affiliations:** Universitat de València, Facultad de Farmacia y Ciencias de la Alimentación, Área de Parasitología, Departamento de Farmacia y Tecnología Farmacéutica y Parasitología, Valencia, Spain

**Keywords:** antimicrobial peptides, *Echinostoma caproni*, helminthiasis, proteomics, susceptibility

## Abstract

**Background:**

Helminth infections are among the most prevalent neglected tropical diseases, causing an enormous impact in health and the socioeconomic status of developing countries. The study host-parasite interactions constitute a promising approach for developing new tools to control these infections. *Echinostoma caproni* is an extensively used model to study host–parasite interactions with emphasis on the factors determining the course of the infections. Herein, we study how infection-induced changes influence host susceptibility and pathology by a proteomic approach.

**Methods:**

Male ICR mice were infected with *E. caproni*. Ileal epithelial cells were isolated 4 weeks post-infection, and proteins were extracted for LC-MS/MS analysis using DDA and SWATH. Differential expression was quantified and analyzed with bioinformatics tools.

**Results:**

The infection induced strong upregulation of proteins involved in mitochondrial oxidative phosphorylation, purine metabolism, and exopeptidases activating the renin–angiotensin system, fostering oxidative stress and inflammation. Downregulated proteins included those linked to fatty acid β-oxidation, nucleotide binding, vesicular trafficking, and tight junction maintenance, indicating impaired epithelial homeostasis. Antimicrobial peptides were overexpressed, while eosinophil- and neutrophil-derived effectors were reduced, suggesting a skewed Th1-biased profile.

**Conclusion:**

The primary *E. caproni* infection in mice triggers profound metabolic and immunological reprogramming, characterized by mitochondrial dysfunction, oxidative stress, and epithelial barrier disruption. These alterations, together with an unbalanced antimicrobial and immune response, shape a pro-inflammatory environment that favors chronic infection. Our findings provide novel insights into the molecular basis of host susceptibility to intestinal helminths and may serve for future therapeutic or preventive strategies.

## 1 Introduction

Intestinal parasitic infections caused by helminths are the most common Neglected Tropical Diseases (NTDs), affecting approximately 1.5 billion people worldwide. Approximately 876 million children worldwide required preventive chemotherapy against soil-transmitted helminthiases in 2023 (1). These infections disproportionately impact populations in low-income countries in Africa, Asia, and Latin America, with children being particularly vulnerable. It is estimated that over 207 million preschool-age and more than 600 million school-age children are infected with one or more species of intestinal helminths, particularly those transmitted through soil (2, 3). However, changes in climatic conditions, globalization, and the movement of people may increase parasite transmission into non-endemic areas (4). Although these infections rarely cause immediate mortality, their public health significance lies in the associated morbidity and significant clinical implications, especially if the infection persists chronically. Parasitic infections can cause intestinal inflammation, diarrhea, malabsorption syndrome, anemia, anorexia, and severe malnutrition and can negatively impact the physical and cognitive development of children and the productivity of adults (5–7). Moreover, beyond their human health impact, intestinal helminths also cause important economic losses in livestock production due to poor weight gain, reduced fertility, and increased veterinary costs (8, 9).

Currently, the control of intestinal helminth infections relies predominantly on the administration of drugs such as albendazole or mebendazole. While these treatments are effective in reducing parasite burden, these drugs are expensive and difficult to implement at the population level. Furthermore, tolerance and the emergence of drug resistance, and above all, the fact that these infections do not generate permanent immunity, make control in the population difficult (10). In light of these limitations, numerous studies have focused on the development of anthelmintic vaccines as a complementary or alternative strategy. However, progress in vaccine development is being very slow and complex, largely due to the fact that the determining factors of resistance or susceptibility to these infections are unknown (11). In this context, various experimental host-parasite models have proven to be useful in advancing the knowledge of host-pathogen interactions and achieving a better comprehension of the factors determining the resistance to intestinal helminths.

*Echinostoma caproni* is an intestinal trematode that has been widely employed as an experimental model to study the factors determining the resistance or susceptibility to intestinal helminth infections. Although *E. caproni* is capable of infecting a wide range of definitive hosts, the degree of host-parasite compatibility varies significantly among rodent species on the basis of worm recovery, fertility, growth, and survival. The differences in worm survival, establishment, and development, make *E. caproni* particularly valuable for comparative studies on the mechanisms associated with resistance and susceptibility to intestinal helminths (12, 13).

Some rodent species, such as rats and hamsters, exhibit a high degree of resistance to *E. caproni* infection, resulting in very short infections, with a limited number of adult worms successfully establishing, and those that do are rapidly eliminated naturally by the host. In contrast, other hosts, such as Institute of Cancer Research (ICR) mice, are highly susceptible to *E. caproni* infection. In these animals, *E. caproni* establishes long-lasting infections characterized by high worm recoveries and prolonged parasite survival. This susceptibility is associated with intense intestinal inflammation and marked tissue damage, which are probably related to the development of a Th1 response. In contrast, resistance to infection in other hosts, such as hamsters and rats, is related to low levels of inflammation, mild pathology and a Th2 type response (14–20).

Previous studies have shown that primary *E. caproni* infections in mice induce a whole series of physiological changes that could be related to the pathology induced by the infection and its chronic development (18). Herein, we extend previous knowledge into the changes induced by primary *E. caproni* infection in a highly susceptible host using an advanced proteomic approach. The aim of this study is to elucidate how infection-induced changes may influence host susceptibility and pathology. The findings are expected to provide insights into the factors that determine the course of intestinal helminth infections.

## 2 Materials and methods

### 2.1 Animals and infection procedures

The present study was performed using male ICR mice weighing 30–35 g. The *E. caproni* strain employed and the infection procedures have been previously described (21). Briefly, encysted metacercariae of *E. caproni* were removed from the kidneys and pericardial cavities of experimentally infected *Biomphalaria glabrata* snails and subsequently used for infection. Four mice were randomly selected, and each was infected by gastric gavage with 25 metacercariae of *E. caproni*. Additionally, the remaining 4 mice were left uninfected and served as controls. All the animals were sacrificed at 4 weeks post-infection for the collection of tissue samples. This time point was selected since it is the time it takes for the worms to reach maturity, the time when the expulsion of worms begins in resistant hosts, and to allow comparisons with previous studies. This fact does not discard that additional changes can be observed at other time points.

### 2.2 Cell collection and protein extraction

Ileal sections from both uninfected and *E. caproni*-infected mice were removed at necropsy, and intestinal epithelial cells were isolated as previously described by Muñoz-Antoli et al. (22). Briefly, the ileal segments were then opened longitudinally and gently rinsed with ice-cold Hank’s Balance Salt Solution supplemented with 2% heat-inactivated fetal calf serum. The supernatant was removed and replaced with fresh washing buffer. This step was repeated at least 4 times until the solution remained clear. Subsequently, the tissue was cut into small, 1 cm-long, segments and incubated for 20 min at 37 °C in a dissociation buffer of HBSS containing 10% fetal calf serum, 1 nM EDTA, 1 mM DTT, 100 U/mL penicillin, and 100 μg/mL streptomycin. The supernatant was collected and kept on ice, and the incubation was repeated with a fresh aliquot of dissociation buffer. The resulting supernatants were combined and passed through a 100 μm cell strainer. Intestinal epithelial cells were then pelleted by centrifugation at 200 g for 10 min at 4 °C and subsequently washed 3 times in PBS under the same conditions to eliminate residual buffer components. For protein extraction, each gram of processed tissue was placed in a new 50 mL Falcon tube containing 15 mL of T-PER reagent, supplemented with 2.5 mL of protease inhibitor cocktail (ethylenediaminetetraacetic acid or EDTA, Roche) and 17.5 µl of phosphatase inhibitor (Sigma Aldrich), to yield final concentrations of 1X and 0.1%, respectively. Tissue homogenization was achieved through alternating cycles of vigorous vortexing and vertical rocking, repeated 3 times for 5 minutes each. The resulting homogenates were transferred to 15 mL Falcon tubes and centrifuged at 5000 g for 5 min at 4 °C. The supernatants were then transferred to new Falcon tubes and subjected to a second centrifugation step under the same conditions. Finally, the clarified protein extracts were aliquoted into 1.5 mL Eppendorf tubes and stored for subsequent analyses.

### 2.3 Differential protein expression analysis in mice infected by tandem mass spectrometry

Differential protein expression in the 8 samples corresponding to the two experimental groups was analyzed using Liquid Chromatography-tandem Mass Spectrometry (LC-MS) employing two complementary approaches: Data-Dependent Acquisition (LC-MS/DDA) and data-independent acquisition using Sequential Window Acquisition of all Theoretical Spectra (LC-MS/SWATH). The initial phase of the analysis involved the construction of a spectral library, accomplished through DDA. This method facilitates the preliminary identification of proteins by selectively fragmenting the most abundant ions detected during the run. Subsequently, SWATH was employed to perform untargeted analysis. This approach allows for enhanced quantification, as it includes data from both high- and low-abundance peptides. The combination of DDA and SWATH acquisition methodologies provides a robust framework for accurate protein identification and reproducible quantification, ensuring comprehensive proteomic profiling of the samples under study (23, 24).

### 2.4 Sample preparation and spectral library construction for LC-MS/MS analysis in DDA and SWATH modes

To identify and quantify proteins present in biological samples from control and *E. caproni*-infected mice, aliquots containing equivalent amounts of protein extract were prepared for analysis. For spectral library construction, 100 µg of representative proteins from each experimental group were pooled. These pooled proteins were subsequently separated by SDS-PAGE, sectioned, and subjected to in-gel enzymatic digestion using sequencing-grade trypsin (Promega). Digestion was carried out with 500 ng of trypsin in ammonium bicarbonate solution at 37 °C. The reaction was terminated by the addition of 10% trifluoroacetic acid (TFA), and peptides were extracted using pure acetonitrile (ACN), dried under vacuum, and resuspended in 20 µL of a 2% ACN/0.1% TFA.

The samples from both groups were randomized previous to proteomic analysis to avoid possible batch effects. For LC-MS/DDA, 5µL of resulting peptide solution were injected into a trapping column (3 μm particle size C18-CL, 120 Ᾰ, 350 μm diameter × 0.5 mm long; Eksigent), desalted with 0.1% TFA for 3 min at a flow rate of 5µL/min, and then separated using an analytical column (3 μm particle size C18-CL, 120 Ᾰ, 75 μm diameter × 150 mm long; Eksigent). Elution was carried out with a linear gradient of ACN (7–40%) in 0.1% formic acid (FA) at 300 nL/min for 60 min. Survey MS1 scans were acquired from 350–1250 m/z for 250 ms. The quadrupole resolution was set to ‘UNIT’ for MS2 experiments, which were acquired 100–1500 m/z for 150 ms in ‘high sensitivity’ mode. Following switch criteria were used: charge of 2+ to 5+ and minimum intensity of 70 counts per second. Up to 25 ions were selected for fragmentation after each survey scan. Dynamic exclusion was set to 15 s.

For individual SWATH analysis, 20 µg of protein extract per sample were processed using the same SDS-PAGE and digestion protocol as described above. 5 µL of peptides were also injected, which were desalted and separated under similar chromatographic conditions, with a gradient of 7–37% ACN in 0.1% FA, at a flow rate of 300 nL/min. The tripleTOF was operated in swath mode, in which a 0.050‐s TOF MS scan from 350–1250 m/z was performed. After, 0.080‐s product ion scans in 100 variable windows from 400 to 1250 m/z were acquired throughout the experiment. The total cycle time was 2.79 s.

Analyses were performed using an Eksigent nanoLC 425 system coupled to a 6600plus TripleTOF spectrometer (ABSCIEX), operated in either DDA or SWATH mode, as appropriate.

### 2.5 Protein identification and quantification from LC-MS/MS data

Protein identification was carried out using the ProteinPilot v5.0 search engine (ABSCIEX), employing the Paragon algorithm (25), which enables the analysis of the wiff files generated by the 6600 TripleTOF mass spectrometer. A series of specific parameters were used, including trypsin specificity, iodoacetamide alkylation of cysteines, searching against the SwissProt database (with and without taxonomic restriction to *Mus musculus*), and 1% false discovery rate (FDR) correction applied at peptide and protein levels. Protein clustering was subsequently performed using the Pro Group algorithm, which organizes identifications based on spectral evidence. For quantification, the wiff files obtained from the SWATH analysis were analyzed using PeakView 2.2 software, endogenous peptides were used for Retention Time calibration to align retention times. Predefined extraction parameters were applied for peptide selection, and protein areas were normalized based on the total signals quantified per sample. The resulting data were exported and statistically processed using various approaches to assess differential protein expression.

### 2.6 Statistical analysis

For the statistical analysis of proteomic data, a penalized multiple regression model was applied. This approach facilitates the construction of robust predictive models to identify and explore biological relationships from omics data exhibiting high dimensionality. Testing included an Elastic Net penalized regression, Principal Component Analysis (PCA), and supervised classification through Partial Least Squares-Discriminant Analysis (PLS-DA). Upon dimensionality reduction, statistically significant biological changes were identified using the regression model implemented via the *Limma* package within the R software (R script in **Supplementary File 1**). A multiple testing correction was performed using the Benjamini and Hochberg procedure to reduce the number of false positives. Resulting adjusted p-values ≤ 0.05 were selected.

Following the identification of the most statistically significant alterations in protein expression, a series of bioinformatics analyses were conducted to uncover biological processes and signaling pathways either activated or repressed during *E. caproni* infection.

Ontology enrichment analysis, specifically Over Representation Analysis (ORA), was used to compare differentially expressed genes or proteins against curated reference gene sets (26). Proteins found to be significantly upregulated (log_2_ fold change _2_ 0) or downregulated (log_2_ fold change < 0) under both experimental conditions were categorized into three Gene Ontology (GO) domains: molecular function (MF), biological processes (BP), and cellular components (CC). These analyses were performed using Bioconductor’s Clusterprofiler, an omics data analysis library specifically for the R software. Statistical significance was assessed using the hypergeometric test followed by Benjamini and Hochberg correction to control the FDR.

Additionally, univariate functional class scoring was performed using Gene Set Enrichment Analysis (GSEA), which allows the examination of expression patterns across the entire dataset in order to identify sets of proteins that are coordinately enriched (27). This method is based on the hypothesis that both large and modest changes in gene or protein expression can significantly impact certain signaling or metabolic pathways. GSEA generates ranked lists of the processes at the level of both GO terms and biological pathways, using GO and Kyoto Encyclopedia of Genes and Genomes (KEGG) as reference databases, respectively. Statistical significance was estimated through gene-ranking permutations to obtain nominal p-values, which were subsequently adjusted for multiple testing using Benjamini and Hochberg method. The Normalized Enrichment Score (NES) was also calculated to enable comparisons across gene sets of different sizes. Only the top 20 enriched GO terms were selected based on the highest NES values. These analyses were also conducted using Bioconductor’s Clusterprofiler.

Finally, a protein-protein interaction network analysis was performed using the open-access platform STRING. This analysis enabled a visual and dynamic understanding of individual and global protein functions based on experimental data, co-expression, or specific databases (28). The parameters used for this analysis followed STRING’s default configuration, including minimum required interaction score, the full STRING network and clustering assignment. Clusters of interacting proteins were determined using a k-means clustering algorithm implemented within STRING. Specifically, protein-protein interaction networks were generated using the proteins associated with the top 20 GO terms identified through GSEA.

### 2.7 Ethics statement

The animals were maintained under conventional conditions with food and water *ad libitum*. This study has been approved by the Ethical Committee of Animal Welfare and Experimentation of the University of Valencia (Ref#A18348501775). Protocols adhered to Spanish (Real Decreto 53/2013) and European (2010/63/UE) regulations.

## 3 Results

### 3.1 Infection rates

The results confirm that mice serve as susceptible hosts to primary *E. caproni* infections, as evidenced by the high infection rates and substantial worm recovery. All mice experimentally challenged with *E. caproni* metacercariae became infected. Worm recovery ranged from 12 to 25 worms per animal, with a mean of 19.3 ± 4.3 (82.4% of worm recovery).

### 3.2 Identification of proteins differentially expressed

A total of 2,196 proteins were identified in the peptide spectral library with a false discovery rate (FDR) of 1% following alignment with the Swissprot database. Subsequent analysis using a database restricted to *M. musculus* yielded 2,282 identified proteins, also with an FDR of 1%. Of these, 1,809 proteins were reliably quantified across the 8 samples corresponding to the control and primary infection groups, with an FDR below 1%.

**Figure 1:**
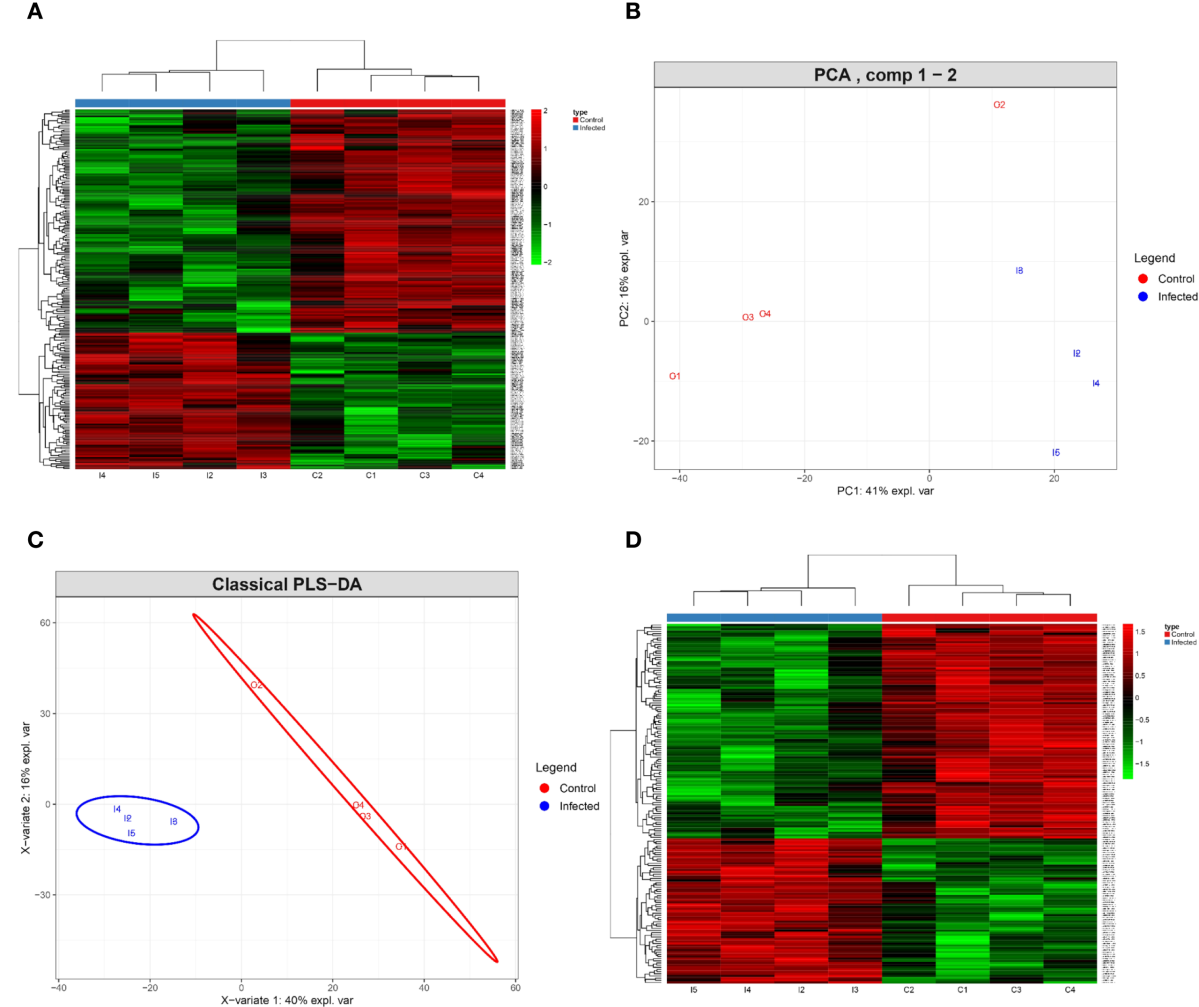
Identification of differentially expressed proteins of intestinal epithelial cells of ICR mice exposed to primary *Echinostoma caproni* infection and control mice. Heatmap showing the hierarchical clustering of 237 proteins from control and infected mice **(A)**. Proteins were subsequently analyzed by principal component analysis (PCA) **(B)** and supervised classification by partial least squares discriminant analysis (PLS-DA) **(C)**, showing that a total of 107 proteins exhibit a Variable Importance in Projection (VIP) > 1.5 and an adjusted p-value < 0.05. These proteins are reflected in the heatmap **(D)**, which defines two main groups of differential protein expression between control and infected mice. The vertical clustering shows similarities between experimental groups, while the horizontal clustering reveals the relative abundance of the identified proteins (red: overexpressed; green: underexpressed).

**Figure 2:**
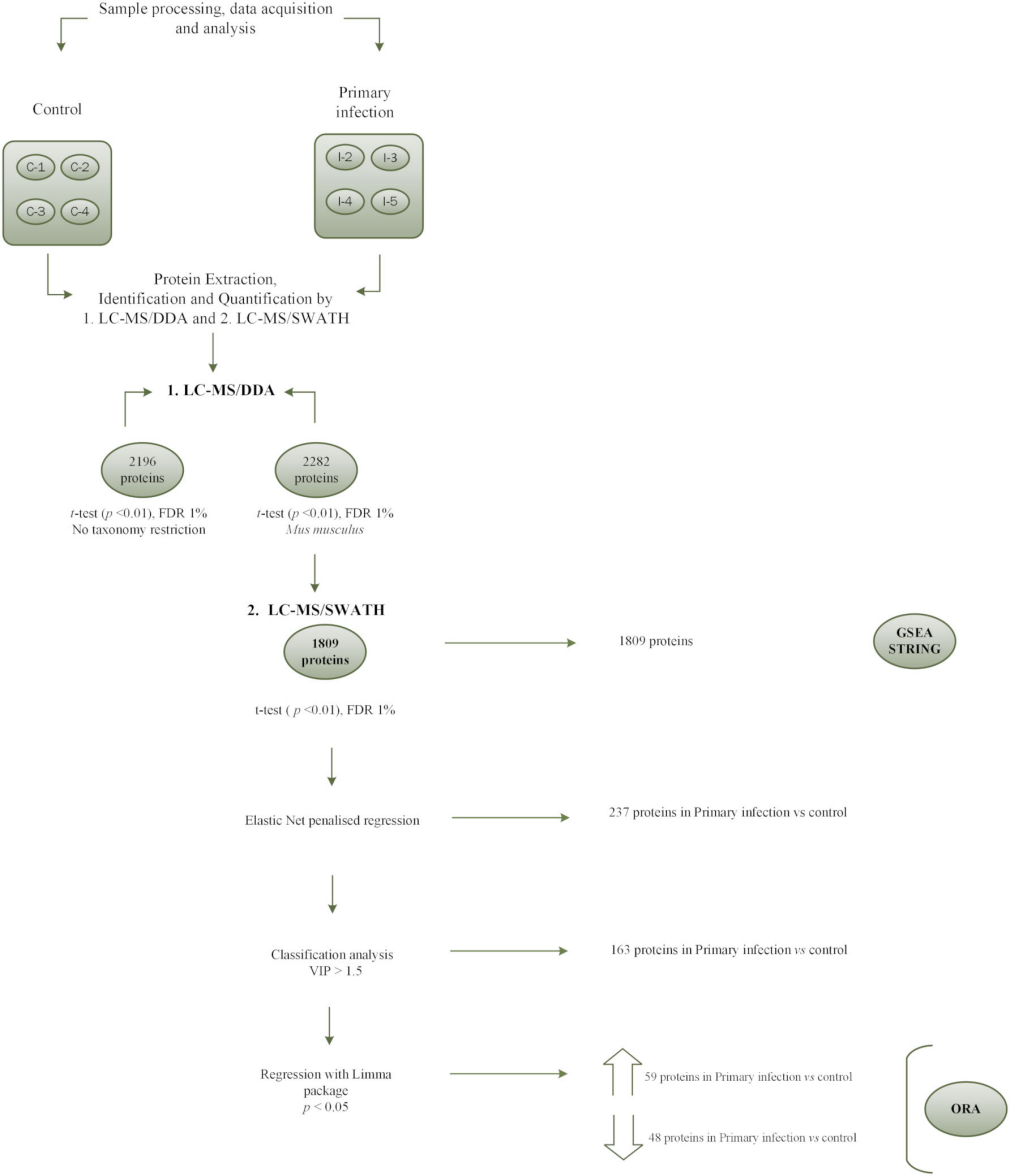
Schematic view of the results obtained in the comparison of protein production profiles of intestinal epithelial cells isolated from controls and infected mice with *Echinostoma caproni*. Four biological samples from control ICR mice (C1-C4) and *E. caproni*-infected mice (I2-I5) were used. A total of 1809 proteins were identified and analyzed using a multiple regression model with Elastic Net model, followed by classification analysis by Variable Importance in Projection (VIP) > 1.5 and regression with the Limma package. Finally, bioinformatics tools were used to allow biological and functional interpretation of the data.

To detect statistically significant differences in protein expression between the two experimental groups, the ElasticNet regularized regression model was applied using optimized parameters via cross-validation using the function *train* with the *caret* package of R software (λ = 0.04 and α = 0.2). The proteins selected by this model are presented in a heatmap (**Figure 1A**), which displays clear segregation of the biological samples according to experimental condition. This clustering effectively discriminated between the control and the infected group, enabling robust identification of differentially expressed proteins. PCA revealed a clear separation between the two groups. The first principal component (PC1) accounted for 41% of the total variance, while the second component (PC2) explained a further 16%, together capturing 57% of the overall variability (**Figure 1B**). Samples from the control group (depicted in red) showed greater dispersion, whereas samples from the infected group (blue) are clustered closely. To further explore discrimination between groups, PLS-DA was performed. This analysis explained 40% and 16% of the variability in the first two components, respectively (**Figure 1C**), confirming the clustering pattern observed in the PCA. Under this supervised approach, the infected group also grouped more coherently. These results provide further evidence of distinct proteomic profiles between the experimental conditions. A second heat map was constructed using only the proteins selected via the Elastic Net model with a variable importance in projection (VIP) value greater than 1.5 in the PLS-DA classification. Two primary clusters were identified, confirming a consistent distinction between control and infected samples based on differential protein abundance (**Figure 1D**). In total, 237 proteins were selected by the Elastic Net model. Of these, 163 proteins exhibited a VIP value greater than 1.5, indicating strong discriminatory power between conditions. A subset of 107 proteins—comprising 59 upregulated and 48 downregulated proteins—reached statistical significance (adjusted p < 0.05) following the Benjamini and Hochberg correction (**Supplementary Table S1**). A schematic representation of the analytical workflow used to identify these proteins is shown in **Figure 2**, while **Table 1** presents the top 20 differentially expressed proteins, ranked by the magnitude of fold change/log_2_ fold change greater or lesser than 0.

**Table 1:** Proteins identified by mass spectrometry as differentially expressed between intestinal epithelial cells of control mice ICR *versus* exposed to primary infection by *Echinostoma caproni*.

ID UniProt	Protein	Fold change	Score	Coverage	Peptides
Q64339	Ubiquitin-like protein ISG15	+5.51	6.02	17.39	4
P51162	Gastrotropin	+5.33	19.74	67.19	11
Q8K1F9	Lactase-like protein	+4.60	2	1.24	1
Q08731	Lithostatine-2	+4.49	2.07	6.94	1
P97449	Aminopeptidase N	+3.92	89.37	48.76	55
P17534	α-defensin-related sequence 2	+3.92	2.22	20.88	2
P35230	Regenerating islet-derived protein 3-β	+3.31	8	29.71	6
P55050	Fatty acid – binding protein, intestinal	+3.15	24.59	56.06	23
P11928	2’-5’-oligoadenylate synthase 1A	+2.78	3.33	11.99	1
Q8R0I0	Angiotensin-converting enzyme 2	+2.77	6.21	8.2	2
Q3UW68	Calpain-13	-3.14	11.07	13.38	8
Q64213	Splicing factor 1	-3.28	7.21	7.5	2
Q2KHK6	Gasdermin-C2	-3.51	18.73	27.5	12
P62751	60S Ribosomal protein L23a	-3.62	12.13	28.85	6
P97425	Eosinophil cationic protein 2	-3.65	6.01	49.36	10
P01794	Ig heavy chain V region HPCG14	-3.91	4.34	26.02	3
P15119	Mast Cell Protease 2	-5.6	18.36	62.3	12
Q80ZA0	Intelectin-1b	-6.11	36.82	58.79	26
P11034	Mast Cell Protease 1	-6.46	23.22	62.6	18
Q64GA5	Cytosolic phospholipase A2 gamma	-7.28	89.59	65.49	59

### 3.3 Functional analysis of omics data with bioinformatics tools

Given the scale and complexity of the observed proteomics changes, functional interpretation was complemented by a combination of ORA, GSEA, and protein-protein interaction network construction via the STRING platform.

#### 3.3.1 Over-representation analysis

Differentially expressed proteins were categorized into three principal GO categories: molecular function (MF), biological processes (BP), and cellular components (CC) (**Supplementary Table S2**).

**Figure 3:**
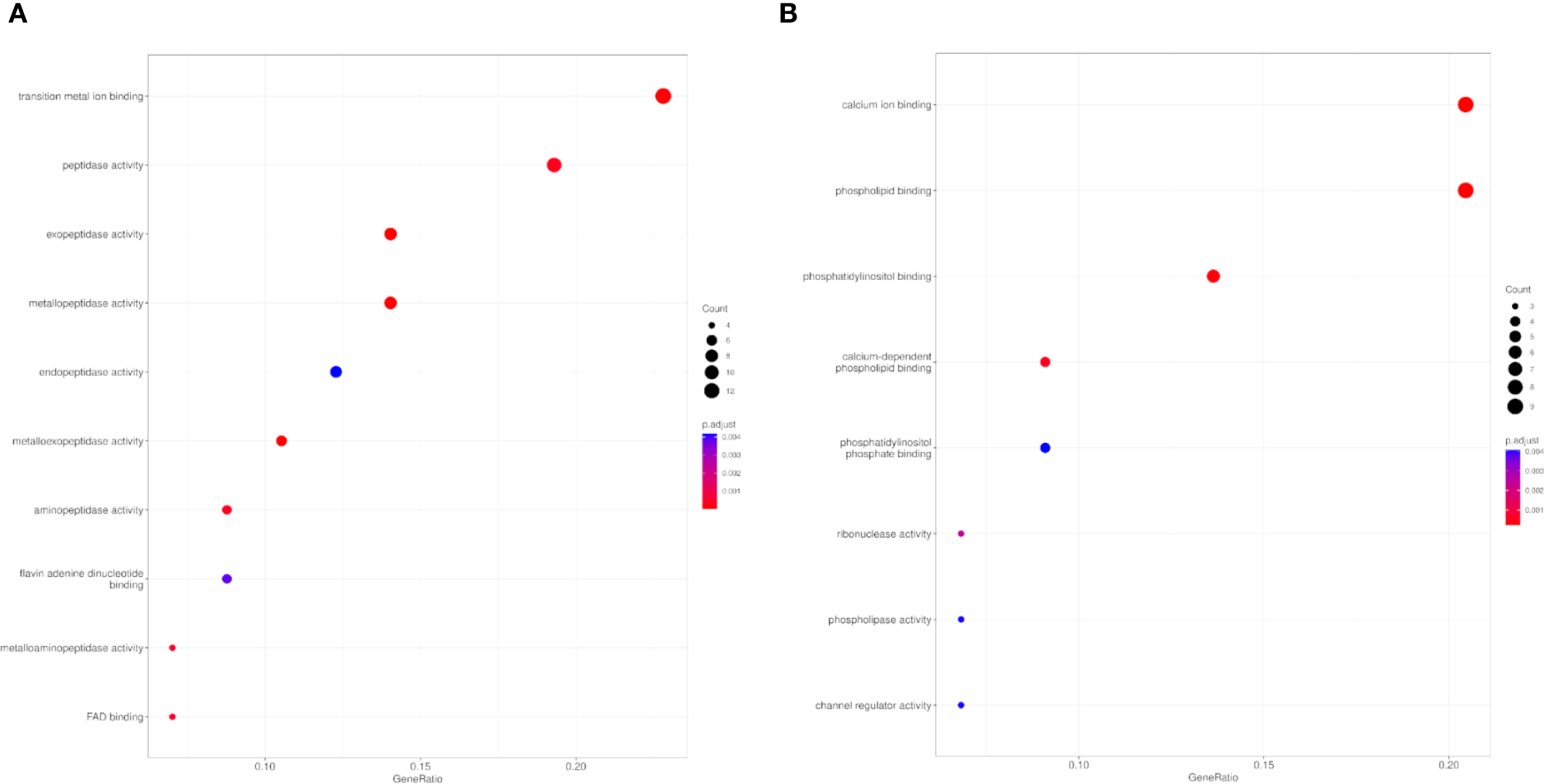
Overrepresentation Analysis (ORA) of molecular function (MF) GO terms of control mice ICR *versus* exposed to primary infection by *Echinostoma caproni*. Top 10 enriched terms in the MF category for overexpressed **(A)** and underexpressed **(B)** proteins in the ileum of the analyzed mice. The size of the dots indicates the number of proteins associated with each term and the color reflects the adjusted p-value.

Primary infection with *E. caproni* was associated with significant over-representation of 17 MF GO terms, predominantly encompassing proteins with hydrolytic activity and metal ion-binding functions (**Table 2**). The most significant terms included exopeptidase activity, metallopeptidase activity, and metalloexopeptidase activity. Additionally, general peptidase activity was also notably enriched (**Figure 3A**). Conversely, only 8 significantly under-expressed FM terms were identified. These predominantly included terms associated with calcium ion binding, phospholipid binding, and phosphatidylinositol binding, all of which are functionally linked to calcium-mediated cell signaling processes. Furthermore, reduced expression was observed for proteins with ribonuclease activity and calcium-dependent phospholipid binding (**Figure 3B**).

**Table 2:** Molecular Function (GO: MF) terms identified by ORA as significantly enriched between intestinal epithelial cells of control mice ICR *versus* exposed to primary infection by *Echinostoma caproni*.

ID	Expression	Description	p-value	Proteins	Number of proteins
GO:0008238	↑	exopeptidase activity	7.88 ^-06^	Anpep/Dpp4/Xpnpep1/Enpep/Ace2/Lap3/Mme/Ace	8
GO:0008237	↑	metallopeptidase activity	2.97 ^-05^	Anpep/Xpnpep1/Enpep/Ace2/Lap3/Mme/Mep1b/Ace	8
GO:0008235	↑	metalloexopeptidase activity	3.21 ^-09^	Anpep/Xpnpep1/Enpep/Ace2/Lap3/Ace	6
GO:0046914	↑	transition metal ion binding	7.22 ^-05^	Anpep/Xpnpep1/Enpep/Lap3/Xdh/Dpyd/Mme/4931406C07Rik/COX2/Mep1b/Ace/Txnrd1/Gda	13
GO:0008233	↑	peptidase activity	0.0003	Anpep/Dpp4/Xpnpep1/Enpep/Psmb10/Ace2/Lap3/Psmb8/Mme/Mep1b/Ace	11
GO:0004177	↑	aminopeptidase activity	0.0004	Anpep/Dpp4/Xpnpep1/Enpep/Lap3	5
GO:0070006	↑	metalloaminopeptidase activity	0.0004	Anpep/Xpnpep1/Enpep/Lap3	4
GO:0071949	↑	FAD binding	0.0006	Xdh/Dpyd/Txnrd1/Acox1	4
GO:0050660	↑	flavin adenine dinucleotide binding	0.0038	Maoa/Xdh/Dpyd/Txnrd1/Acox1	5
GO:0004175	↑	endopeptidase activity	0.0041	Dpp4/Psmb10/Ace2/Psmb8/Mme/Mep1b/Ace	7
GO:0005509	↓	calcium ion binding	0.0002	Pla2G4C/Itlnb/Capn13/Pnliprp2/Anxa1/Anxa3/S100A9/Capn2/Pla2g4a	9
GO:0005543	↓	phospholipid binding	0.0002	Pla2g4c/Tln1/Anxa1/Gsdmc2/Ncf1/Anxa3/Pla2g4a/Snx12/Osbp	9
GO:0035091	↓	phosphatidylinositol binding	0.0003	Tln1/Gsdmc2/Ncf1/Pla2g4a/Snx12/Osbp	6
GO:0005544	↓	calcium-dependent phospholipid binding	0.0006	Pla2g4c/Anxa1/Anxa3/Pla2g4a	4
GO:0004540	↓	ribonuclease activity	0.0024	Rexo2/Ang4/Ear2	3
GO:0004620	↓	phospholipase activity	0.0040	Pla2g4c/Pnliprp2/Pla2g4a	3
GO:0016247	↓	channel regulator activity	0.0040	Gpd1l/Atp2b4/Cd44	3
GO:1901981	↓	phosphatidylinositol phosphate binding	0.0040	Gsdmc2/Pla2g4a/Snx12/Osbp	4

↑, Overexpressed terms; ↓, Underexpressed terms.

**Figure 4:**
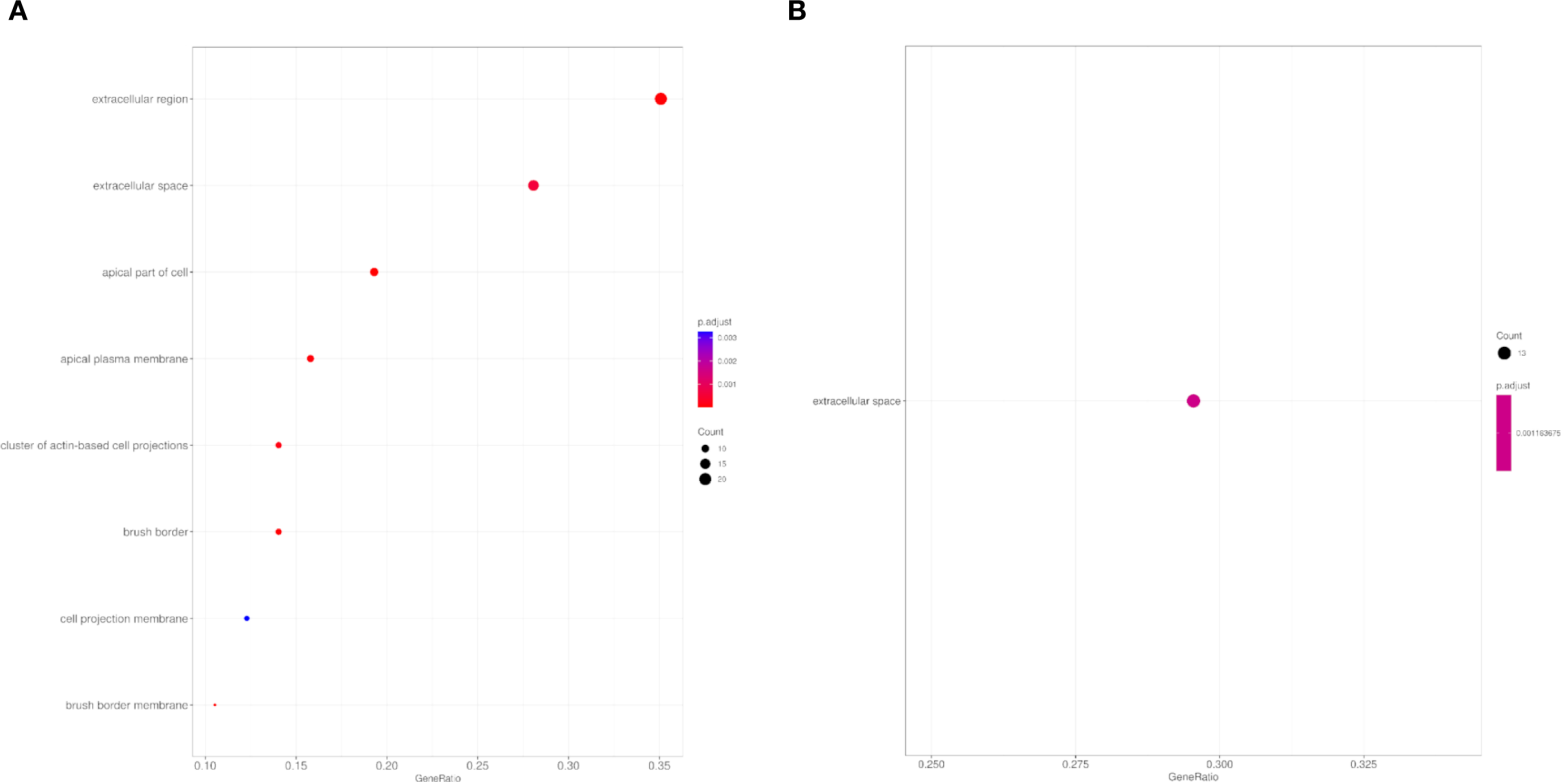
Overrepresentation Analysis (ORA) of biological processes (BP) GO terms of control mice ICR *versus* exposed to primary infection by *Echinostoma caproni.* Top 10 enriched terms in the BP category for overexpressed **(A)** and underexpressed **(B)** proteins in the ileum of the analyzed mice. The size of the dots indicates the number of proteins associated with each term and the color reflects the adjusted p-value.

**Figure 4** provides a graphical representation of the results obtained from the ORA analysis for the BP category, while detailed data regarding both over- and under-expressed GO terms are summarized in **Table 3**. Primary *E. caproni* infection resulted in the significant overexpression of 97 GO terms, most of which were related to antimicrobial defense mechanisms. Among the most significantly enriched BPs were defense response to bacteria, antimicrobial peptide-mediated humoral immune response, antimicrobial humoral response, and regulation of lymphocyte-mediated immunity (**Figure 4A**). Conversely, a marked under-expression was observed for proteins involved in broader immune responses and metabolic functions. A total of 25 GO terms were identified, including defense response, interspecies interactions between organisms, response to external biotic stimuli, and catabolic processes of organic compounds (**Figure 4B**).

**Table 3:** Biological Processes (GO: PB) terms identified by ORA as significantly enriched between intestinal epithelial cells of control mice ICR *versus* exposed to primary infection by *Echinostoma caproni*.

ID	Expression	Description	p-value	Proteins	Number of proteins
GO:0061844	↑	antimicrobial humoral immune response mediated by antimicrobial peptide	8.76 ^-06^	Defa-rs2/Reg3b/Lgals4/B2m/Reg2/Dmbt1	6
GO:0042742	↑	defense response to bacterium	3.25 ^-05^	Isg15/Oas1a/Defa-rs2/Reg3b/Lgals4/B2m/Dmbt1/Lgals9	8
GO:0019730	↑	antimicrobial humoral response	3.47 ^-05^	Defa-rs2/Reg3b/Lgals4/B2m/Reg2/Dmbt1	6
GO:0043171	↑	peptide catabolic process	7.33 ^-05^	Anpep/Xpnpep1/Enpep/Mrs/Ace	5
GO:0009264	↑	deoxyribonucleotide catabolic process	0.0001	Pnp/Xdh/Dpyd/Gda	4
GO:0019731	↑	antibacterial humoral response	0.0001	Defa-rs2/Lgals4/B2m/Dmbt1	4
GO:0002706	↑	regulation of lymphocyte-mediated immunity	0.0002	Dpp4/B2m/Ceacam1/Pnp/Lgals9	5
GO:0003073	↑	regulation of systemic blood pressure	0.0002	Enpep/Ace2/Plcb3/Ace	4
GO:0009162	↑	deoxyribonucleotide monophosphate metabolic process	0.0002	Pnp/Xdh/Dpyd/Gda	4
GO:1901658	↑	glycosyl compound catabolic process	0.0002	Pnp/Xdh/Dpyd/Gda	4
GO:0042742	↓	defense response to bacterium	5.97 ^-05^	Prg2/Gsdmc2/Ncf1/Ang4/Epx/Ear2/Anxa3	7
GO:0009617	↓	response to bacteria	7.13 ^-05^	Prg2/Vim/Pnliprp2/Gsdmc2/Ncf1/Ang4/Epx/Ear2/Anxa3/S100a9	10
GO:0046470	↓	phosphatidylcholine metabolic process	0.0001	Pla2g4c/Pnliprp2/Capn2/Pla2g4a	4
GO:0006952	↓	defense response	0.0001	Itlnb/Prg2/Vim/Pnliprp2/Anxa1/Gsdmc2/Ncf1/Ang4/Epx/Ear2/Anxa3/S100a9/Lgals1/Pla2g4a/Cd44	15
GO:0098542	↓	defense response to another organism	0.0005	Itlnb/Prg2/Vim/Anxa1/Gsdmc2/Ncf1/Ang4/Epx/Ear2/Anxa3/S100a9	11
GO:0044419	↓	biological process involved in interspecies interaction between organisms	0.0006	Itlnb/Prg2/Rab5a/Vim/Pnliprp2/Anxa1/Gsdmc2/Ncf1/Ang4/Epx/Ear2/Anxa3/S100a9/Lgals1	14
GO:0045017	↓	glycerolipid biosynthetic process	0.0007	Pla2g4c/Ang4/Capn2/Pla2g4a	4
GO:0008654	↓	phospholipid biosynthetic process	0.0014	Pla2g4c/Capn2/Pla2g4a/Osbp	4
GO:0043207	↓	response to external biotic stimulus	0.0015	Itlnb/Prg2/Vim/Pnliprp2/Anxa1/Gsdmc2/Ncf1/Ang4/Epx/Ear2/Anxa3/S100a9	12
GO:0051707	↓	response to another organism	0.0015	Itlnb/Prg2/Vim/Pnliprp2/Anxa1/Gsdmc2/Ncf1/Ang4/Epx/Ear2/Anxa3/S100a9	12

↑, Overexpressed terms; ↓, Underexpressed terms.

**Figure 5:**
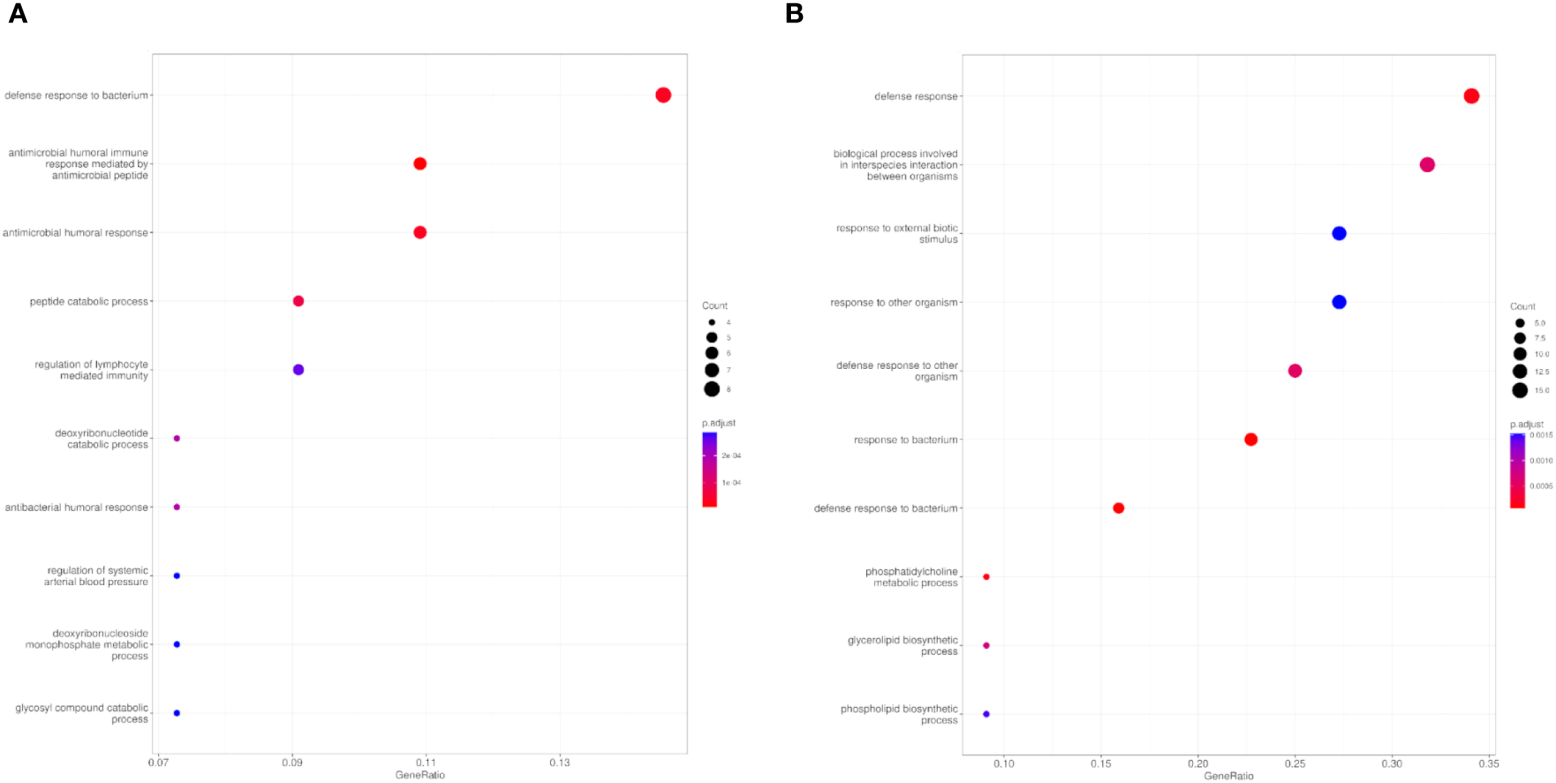
Overrepresentation Analysis (ORA) of cellular components (CC) GO terms of control mice ICR *versus* exposed to primary infection by *Echinostoma caproni*. Top 10 enriched terms in the CC category for overexpressed **(A)** and underexpressed **(B)** proteins in the ileum of the analyzed mice. The size of the dots indicates the number of proteins associated with each term and the color reflects the adjusted p-value.

The results of the ORA analysis for the CC category are illustrated in **Figure 5**, with further details presented in **Table 4**. A total of 9 GO terms were significantly enriched among the overexpressed proteins, mainly associated with cellular compartments located in the extracellular region and apical domains. Notably enriched terms included extracellular region, extracellular space, apical part of cell, and apical plasma membrane. Additionally, enrichment was also observed for specialized structures such as brush border and brush border membrane (**Figure 5A**). On the other hand, among the underexpressed proteins, only the term extracellular space showed statistically significant enrichment (**Figure 5B**).

**Table 4:** Cellular components (GO: CC) terms identified by ORA as significantly enriched between intestinal epithelial cells of control mice ICR *versus* exposed to primary infection by *Echinostoma caproni*.

ID	Expression	Description	p-value	Proteins	Number of proteins
GO:0031526	↑	brush border membrane	6.96 ^-06^	Anpep/Enpep/Ace2/Cdhr2/Slc5A1/Ace	6
GO:0045177	↑	apical part of the cell	3.71 ^-05^	Dpp4/Enpep/Reg3b/Ceacam1/Atp1B1/Fabp2/Ace2/Cdhr2/Slc5A1/Muc13/Slc3A2	11
GO:0005903	↑	brush border	5.87 ^-05^	Anpep/Enpep/Ace2/Cdhr2/Lctl/Ms/Slc5A1/Ace	8
GO:0005576	↑	extracellular region	5.90 ^-05^	Isg15/Lgals3bp/Anpep/Dpp4/Defa-rs2/Reg3b/Lgals4/B2m/Ceacam1/np/Gpt/Ace2/Xdh/Reg2/Dmbt1/Col15a1/Muc13/Mep1b/Lgals9/Ace	20
GO:0016324	↑	apical plasma membrane	8.74 ^-05^	Dpp4/Enpep/Ceacam1/Atp1b1/Ace2/Cdhr2/Slc5A1/Muc13/Slc3A2	9
GO:0098862	↑	cluster of actin-based cell projections	0.0001	Anpep/Enpep/Ace2/Cdhr2/Lctl/Ms/Slc5A1/Ace	8
GO:0005615	↑	extracellular space	0.0005	Lgals3bp/Anpep/Defa-rs2/Reg3b/Lgals4/B2m/Ceacam1/Gpt/Ace2/Xdh/Reg2/Dmbt1/Col15a1/Muc13/Lgals9/Ace	16
GO:0031253	↑	cell projection membrane	0.0032	Anpep/Enpep/Ceacam1/Ace2/Cdhr2/Slc5A1/Ace	7
GO:0005615	↓	extracellular Space	0.0011	Mcpt1/Mcpt2/Itlnb/Pnliprp2/Ckm/Anxa1/Ang4/Aldoa/Epx/Ear2/S100a9/Lgals1/Dpysl3	13

↑, Overexpressed terms; ↓, Underexpressed terms.

#### 3.3.2 Gene set enrichment analysis

**Figure 6:**
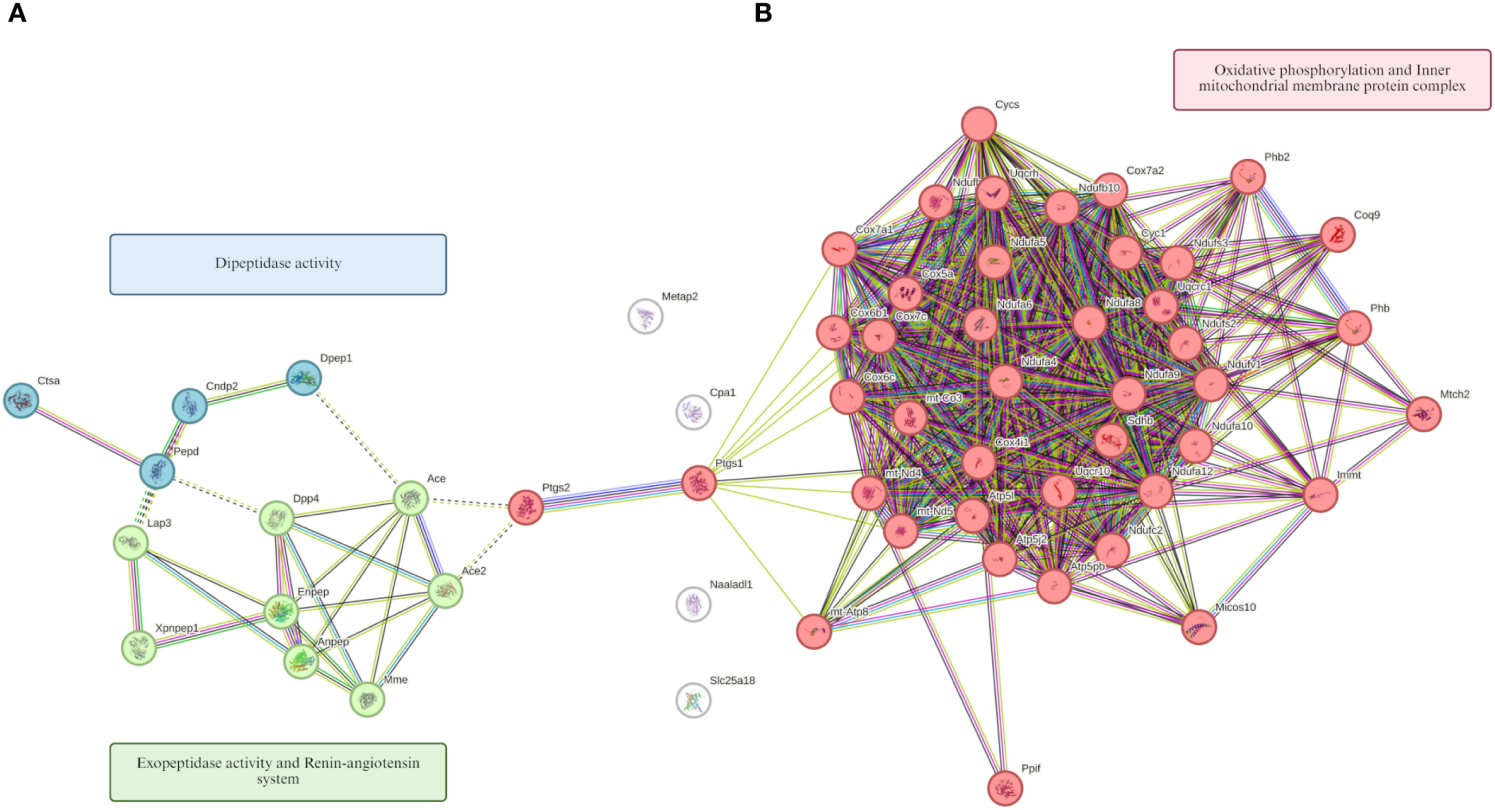
Principal GO terms using gene set enrichment analysis (GSEA) of control mice ICR *versus* exposed to primary infection by *Echinostoma caproni*. Top 10 most representative GO terms associated with overexpressed **(A)** and underexpressed **(B)** proteins in the ileum of the analyzed mice. The size of the dots indicates the number of proteins associated with each term and the color reflects the adjusted p-value.

This analysis revealed 234 significantly overexpressed and 704 underexpressed GO terms (**Supplementary Table S3**). The top 20 most significant enrichment terms in each category are summarized in **Figure 6** and **Table 5**. Among the most overexpressed terms were those associated with aerobic respiration, the electron transport chain, and oxidative phosphorylation. These processes predominantly involved proteins located in the mitochondrial respirasome complex. Additionally, enrichment was also observed for GO terms related to exopeptidase activity (**Figure 6A**). Furthermore, primary *E. caproni* infection led to impaired activation of processes linked to the binding of nucleotides, nucleosides, ribonucleotides, ribonucleosides, carbohydrate derivatives, heterocyclic compounds, cyclic organic compounds, and small molecules (**Figure 6B**).

**Table 5:** Gene Ontology (GO) terms identified by GSEA as significantly enriched between intestinal epithelial cells of control mice ICR *versus* exposed to primary infection by *Echinostoma caproni*.

GO	ID	Expression	Description	ES	NES	p-value	Proteins
CC	GO:0005746	↑	mitochondrial respirasome	0.698	2.55	3.63 ^-09^	ND5/Cox6c/COX2/Cox7a1/Cox7a2/Cox4i1/Ndufs2/Cox5a/Ndufa12…
CC	GO:0070469	↑	respirasome	0.698	2.55	3.63 ^-09^	ND5/Cox6c/COX2/Cox7a1/Cox7a2/Cox4i1/Ndufs2/Cox5a/Ndufa12…
CC	GO:0098803	↑	respiratory chain complex	0.698	2.55	3.63 ^-09^	ND5/Cox6c/COX2/Cox7a1/Cox7a2/Cox4i1/Ndufs2/Cox5a/Ndufa12…
BP	GO:0042773	↑	ATP synthesis coupled electron transport	0.697	2.50	6.87 ^-08^	ND5/Cox6c/Coq9/COX2/Cox7a1/Cox7a2/Cox4i1/Ndufs2/Cox5a/Ndufa12…
BP	GO:0042775	↑	mitochondrial ATP synthesis coupled electron transport	0.69	2.46	1.41 ^-07^	ND5/Cox6c/Coq9/Cox7a1/Cox7a2/Cox4i1/Ndufs2/Cox5a/Ndufa12/COX1…
BP	GO:0019646	↑	aerobic electron transport chain	0.68	2.39	7.79 ^-07^	ND5/Cox6c/Coq9/Cox7a1/Cox7a2/Cox4i1/Ndufs2/Cox5a/COX1/Ndufa10…
MF	GO:0008238	↑	exopeptidase activity	0.661	2.37	2.22 ^-06^	Anpep/Cpa1/Ace2/Enpep/Naaladl1/Dpp4/Xpnpep1/Pepd/Ctsa/Mme/Lap3…
BP	GO:0006119	↑	oxidative phosphorylation	0.579	2.32	3.82 ^-07^	ND5/Cox6c/Coq9/COX2/Ppif/Cox7a1/Cox7a2/Cox4i1/Ndufs2/Cox5a/Ndufa12…
BP	GO:0022904	↑	respiratory electron transport chain	0.582	2.17	4.18 ^-06^	ND5/Cox6c/Coq9/COX2/Cox7a1/Cox7a2/Cox4i1/Ndufs2/Cox5a/Ndufa12…
CC	GO:0098800	↑	inner mitochondrial membrane protein complex	0.595	2.38	3.50 ^-08^	ND5/Cox6c/COX2/Cox7a1/Cox7a2/Cox4i1/Ndufs2/Cox5a/Micos10/Ndufa12…
MF	GO:0032555	↓	purine ribonucleotide binding	-0.548	-2.36	1.00 ^-10^	Aacs/Mcm4/Mcm2/Rab8a/Pfkp/Eif5b/Pkm/Dars/Aprt/Gimap4/Septin2/Cdk11b…
MF	GO:0032553	↓	ribonucleotide binding	-0.547	-2.35	1.00 ^-10^	Aacs/Mcm4/Mcm2/Rab8a/Pfkp/Eif5b/Pkm/Dars/Aprt/Gimap4/Septin2/Cdk11b/…
MF	GO:0035639	↓	purine ribonucleoside triphosphate binding	-0.543	-2.33	1.00 ^-10^	Aacs/Mcm4/Mcm2/Rab8a/Pfkp/Eif5b/Pkm/Dars/Gimap4/Septin2/Cdk11b/Arf3…
MF	GO:0017076	↓	purine nucleotide binding	-0.515	-2.24	1.00 ^-10^	Aacs/Mcm4/Mcm2/Rab8a/Pfkp/Eif5b/Pkm/Dars/Aprt/Gimap4/Septin2/Cdk11b…
MF	GO:0032559	↓	adenyl ribonucleotide binding	-0.501	-2.10	1.00 ^-10^	Aacs/Mcm4/Mcm2/Pfkp/Pkm/Dars/Aprt/Cdk11b/Fcsk/Uba5/Hsp90b1/Farsb…
MF	GO:0000166	↓	nucleotide binding	-0.472	-2.07	1.00 ^-10^	Rab21/Hsp90b1/Farsb/Akr1b8/Fkbp4/Ddx39a/Ero1a/Slk/Atp5a1/Acat1…
MF	GO:1901265	↓	nucleoside phosphate binding	-0.472	-2.07	1.00 ^-10^	Rab21/Hsp90b1/Farsb/Akr1b8/Fkbp4/Ddx39a/Ero1a/Slk/Atp5a1/Acat1…
MF	GO:0097367	↓	carbohydrate derivative binding	-0.46	-2.00	1.00 ^-10^	Aacs/Mcm4/Mcm2/Rab8a/Pfkp/Eif5b/Pkm/Dars/Aprt/Gimap4/Septin2…
MF	GO:0036094	↓	small molecule binding	-0.417	-1.85	1.00 ^-10^	Rab21/Hsp90b1/Farsb/Akr1b8/Fkbp4/Ddx39a/Ero1a/Slk/Atp5a1/Acat1…
MF	GO:1901363	↓	heterocyclic compound binding	-0.405	-1.83	1.00 ^-10^	Rab21/Hsp90b1/Farsb/Akr1b8/Fkbp4/Hnrnpa1/Ddx39a/Ero1a/Trim28…

↑, Overexpressed terms; ↓, Underexpressed term; ES, Enrichment Score; NES, Normalized Enrichment Score.

**Figure 7:**
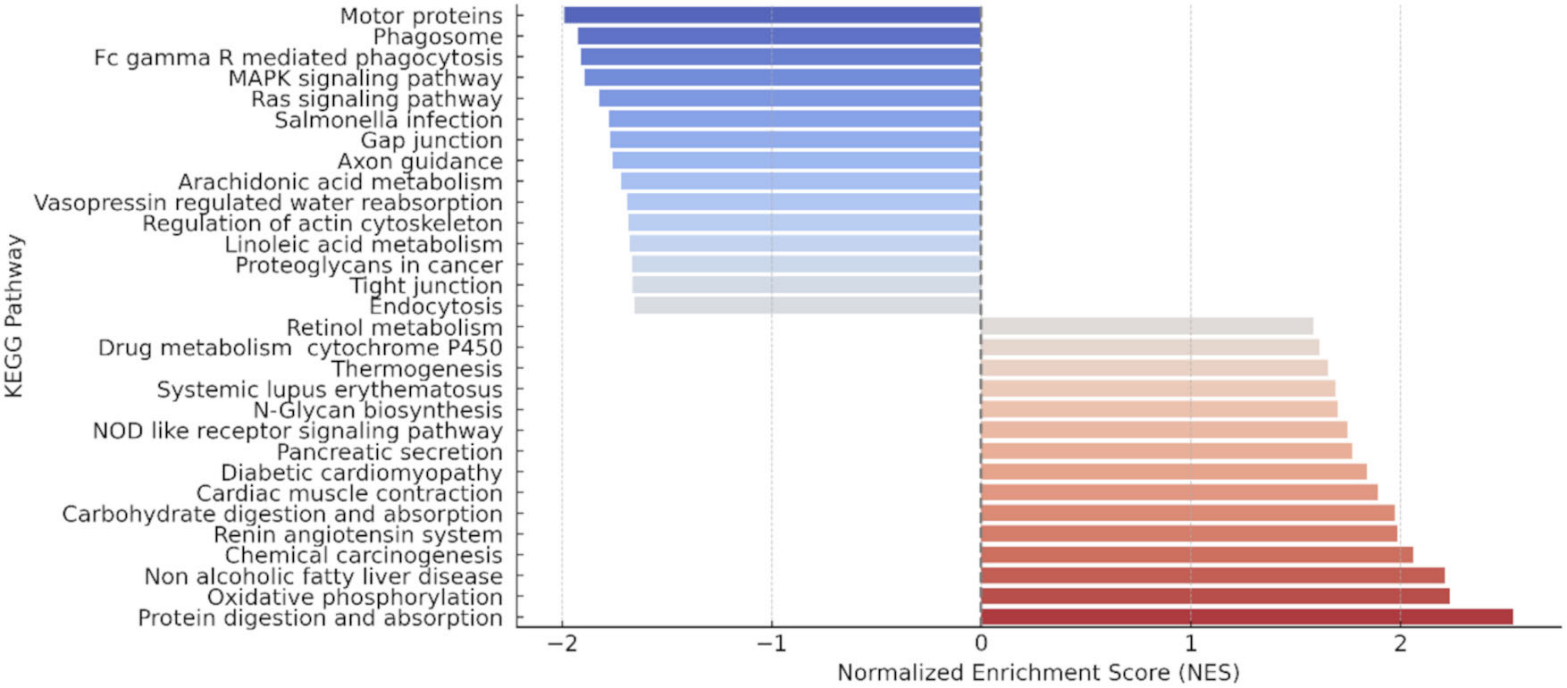
Principal pathways enriched using the kyoto encyclopedia of genes and genomes (KEGG) of control mice ICR *versus* exposed to primary infection by *Echinostoma caproni*. The graph shows the signaling pathways and metabolic processes enriched in the ileum of ICR mice, sorted by their Normalized Enrichment Score (NES) and indicating overactivated (red) and suppressed (blue) pathways.

The database KEGG was used to functionally contextualize the global changes in protein expression observed during primary infection by *E. caproni*. A comparison between the proteomes of infected and non-infected control mice revealed the significant overactivation of 54 biological pathways and the suppression of 58 (**Supplementary Table S3**). Only the main over- and underexpressed terms are shown in **Figure 7**. The results indicate that the infection induces the activation of metabolic processes related to the digestion and absorption of proteins and carbohydrates, oxidative phosphorylation, activation of the renin-angiotensin system, and the NOD-like receptor signaling pathway. Activated pathways related to the biosynthesis of N-glycans and fatty acids were also observed, as well as processes linked to endocannabinoid retrograde signaling and neurodegeneration. Conversely, negatively enriched pathways identified functions related to motor proteins, phagosome formation, MAPK signaling, endocytosis, Fcg-mediated phagocytosis, and the Ras signaling pathway. Furthermore, significant alterations were detected in signaling pathways essential for the maintenance of intestinal epithelial barrier integrity, including gap and tight junctions.

#### 3.3.3 Protein-protein interaction network analysis using STRING

**Figure 8:**
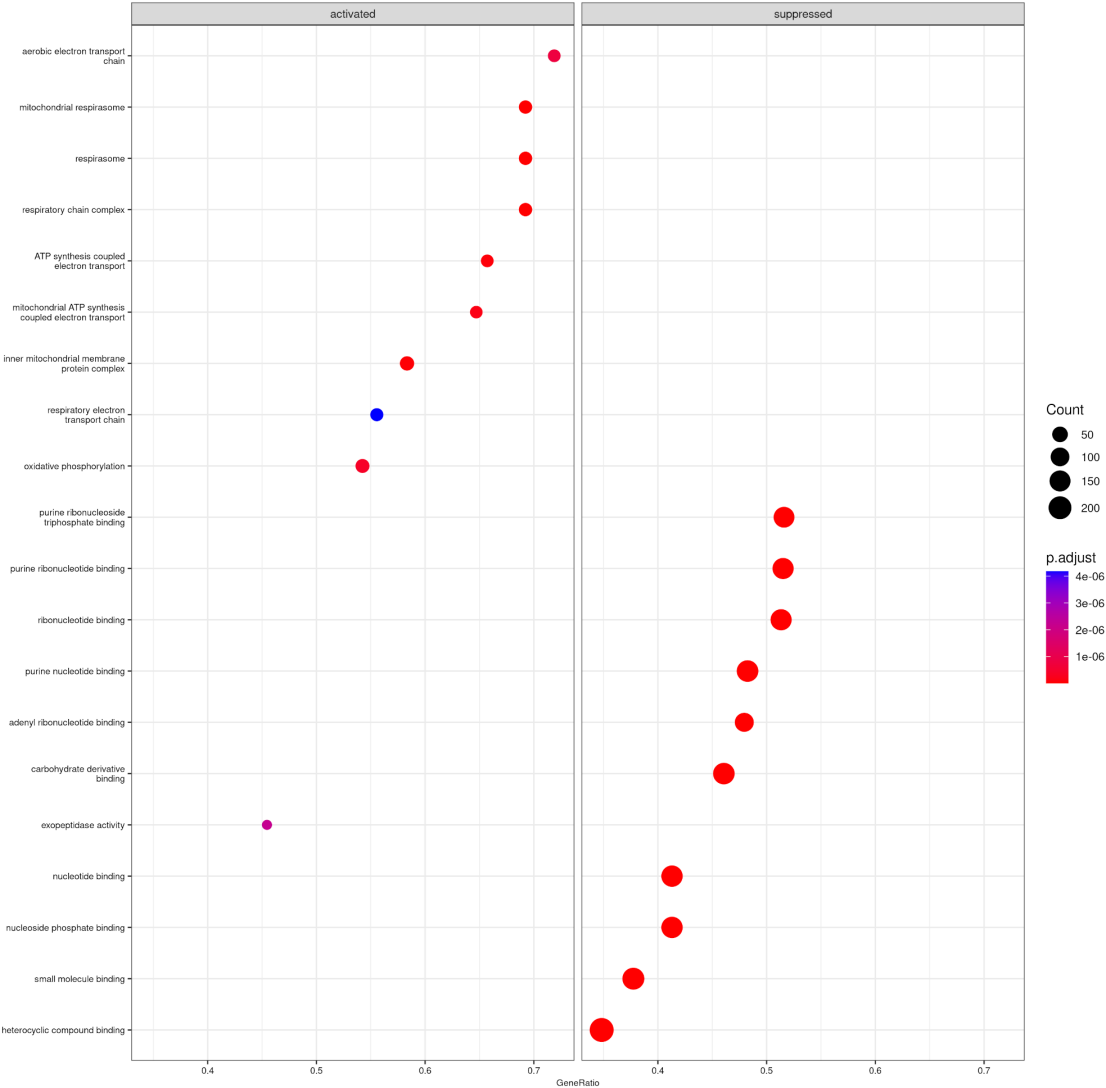
Protein-protein interaction network from overexpressed proteins. Proteins were grouped into the top 10 GO terms using GSEA analysis in the ileum of ICR mice with primary *Echinostoma caproni* infection *versus* control group. Hierarchical grouping of overexpressed proteins into three clusters is observed. Node size represents the relative centrality of each protein in the network and the color reflects the degree of connectivity or interaction density within the group. Lines indicate the type of interaction evidence supporting the connection between nodes (experimental data, coexpression, or curated databases).

**Figure 9:**
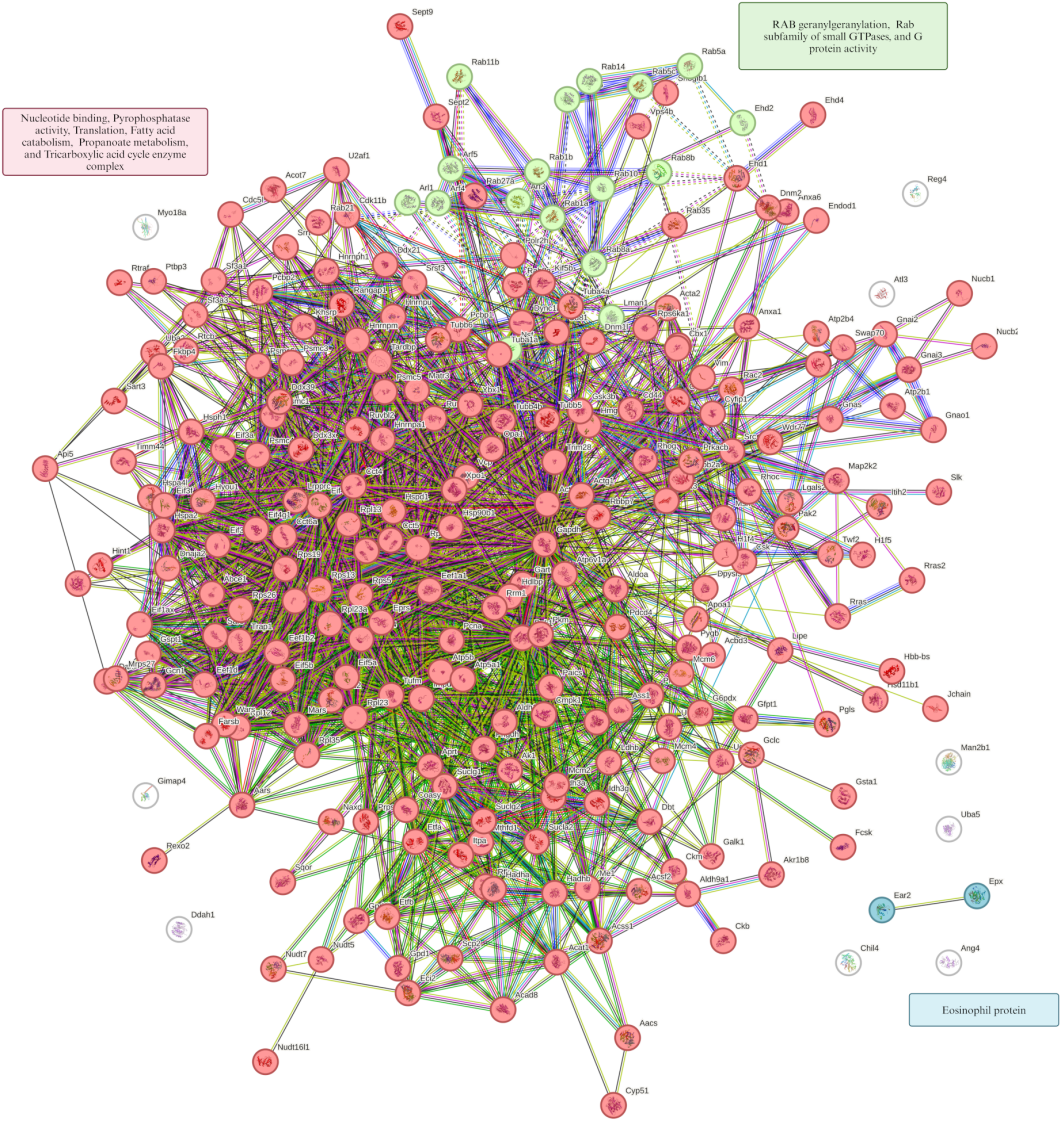
Protein-protein interaction network from underexpressed proteins. Proteins were grouped into the top 10 GO terms using GSEA analysis in the ileum of ICR mice with primary *Echinostoma caproni* infection *versus* control group. Hierarchical grouping of underexpressed proteins into three clusters is observed. Node size represents the relative centrality of each protein in the network and the color reflects the degree of connectivity or interaction density within the group. Lines indicate the type of interaction evidence supporting the connection between nodes (experimental data, coexpression, or curated databases).

This approach enabled the identification of hierarchical protein clusters with related biological functions, providing insight into relevant molecular associations during primary *E. caproni* infection. The subset of proteins corresponding to the most significant over- and underexpressed GO terms was analyzed for functional connectivity. In the group of mice subjected to a primary *E. caproni* infection, in comparison with the uninfected controls, the resulting interaction network revealed three distinct clusters of functionally interconnected proteins (**Figure 8**). The first cluster (red) comprised proteins implicated in oxidative phosphorylation and components of the inner mitochondrial membrane protein complex. The second cluster (green) grouped proteins with exopeptidase activity, mainly linked to activation of the renin-angiotensin system. The third cluster (blue) encompassed proteins exhibiting dipeptidase activity. A parallel protein-protein interaction network was constructed for underexpressed proteins, which also revealed three functional clusters (**Figure 9**). The first (red) grouped proteins related to nucleotide binding, pyrophosphatase activity, translation, fatty acid catabolism, propanoate metabolism, and enzymes involved in the tricarboxylic acid cycle. The second cluster (green) comprised proteins involved in the geranylgeranylation of RAB and members of the Rab subfamily of small GTPases, which are crucial for vesicular transport and intracellular signaling. Finally, the third cluster (blue) consisted of eosinophil-derived proteins known to participate in the immune response against helminth infections.

## 4 Discussion

Our findings indicate that susceptibility to primary *E. caproni* infections in mice is associated with the activation of a complex functional response, characterized by alterations in multiple metabolic, immunological, and epithelial processes triggered by the infection. This of interest, since resistance in other host species is related to milder responses and the activation of mechanisms responsible for the regeneration of the intestinal tissue leading to early expulsion of this helminth (19, 22).

The significant metabolic changes are associated to a sustained oxidative and proinflammatory environment via distinct metabolic pathways, that potentially may contribute to tissue damage and the chronic progression of the infection. Observed metabolic changes were marked by functional enrichment in the overexpression of mitochondrial proteins associated with oxidative phosphorylation, mitochondrial ATP synthesis coupled to the electron transport chain, and the mitochondrial respirasome.

Notable among the identified proteins were isoforms of NADH, cytochrome C oxidases, and ATPases. Mitochondria are primarily responsible for energy generation through several metabolic routes, including β-oxidation of fatty acids, conversion of pyruvate into acetyl-CoA through the tricarboxylic acid cycle and oxidative phosphorylation (29). However, growing evidence suggests that dysregulation of these pathways may adversely affect cellular homeostasis (30, 31).

Furthermore, underexpression of terms associated with nucleotide-binding proteins involved in the tricarboxylic acid cycle also was identified, including isoforms of the mitochondrial isocitrate dehydrogenase subunits and mitochondrial succinate-CoA ligase. In this context, previous studies have reported that the cells deficient in mitochondrial isocitrate dehydrogenase 1 and 2 are susceptible to lipid peroxidation, oxidative DNA damage, and intracellular peroxide generation (32). Simultaneous upregulation of oxidative phosphorylation proteins and downregulation of TCA cycle enzymes may appear contradictory. However, this phenomenon could be explained by several mechanisms. Chronic mitochondrial ROS production during *E. caproni* infection preferentially damages Complex I. Moreover, downregulation of TCA enzymes can limit the supply of NADH and FADH_2_, especially via Complex II (SDH) as a protective response to prevent further ROS generation from a damaged Complex I (“reverse electron transport” mitigation). Finally, the mitochondrion may respond by increasing the stoichiometric excess of respiratory chain complexes in an attempt to sustain Δψm and residual forward electron flow using whatever reduced substrates are still available.

A downregulation of proteins involved in fatty acid β-oxidation was also observed, including mitochondrial acetyl-CoA acetyltransferase, subunits of the mitochondrial trifunctional enzyme, and mitochondrial isobutyl-CoA dehydrogenase. It has been demonstrated that acetyl-CoA deficiency can lead to exacerbated liver fibrosis in murine models due to the accumulation of free fatty acids in hepatocytes (33). These findings therefore corroborate and extend the observations made by Cortés et al. (18, 34) regarding the complexity of metabolic dysregulation in host intestinal cells during *E. caproni* infection. The present study reveals a novel finding: an overactivation of purine deoxyribonucleotide metabolism, suggesting a compensatory mechanism for ATP production in a context of partially dysregulated mitochondrial activity. Enzymes identified within this pathway included dihydropyrimidine dehydrogenase, purine nucleoside phosphorylase, guanine deaminase, and xanthine dehydrogenase/oxidase. This observation could be relevant, as current evidence suggests that ATP hydrolysis products (ADP, AMP, IMP, inosine, and hypoxanthine) are essential substrates for ATP synthesis via oxidative phosphorylation (35). Moreover, this alternative metabolic route could also have pathophysiological consequences. Both oxidative phosphorylation and purine degradation are associated with increased production of reactive oxygen species, generated as a product of the electron transfer along the mitochondrial respiratory chain (36, 37). In particular, xanthine dehydrogenase/oxidase has been linked to the generation of uric acid, hydrogen peroxide, and oxygen free radicals, all of which may contribute to increased oxidative stress (38). This aspect, not previously explored in experimental *E. caproni* infection, suggest a potentially critical pathway in the pathophysiology of chronic infection.

Additionally, functional enrichment was identified in the overexpression of extracellular exopeptidases involved in both the activation of the renin-angiotensin system and the digestion and absorption of molecules. Among the overexpressed proteins, notable examples include isoforms of angiotensin-converting enzyme and prostaglandin endoperoxide synthase, as well as various aminopeptidases, metallopeptidases, and carboxypeptidases. Of particular interest, the present study revealed an overexpression of angiotensin-converting enzyme 2 (ACE2, + 2.77), an enzyme capable of activating NADHP to promote reactive oxygen species production in smooth muscle and endothelial cells following tissue injury (39). These findings reinforce existing evidence that renin-angiotensin system activation plays a central role in amplifying inflammation and promoting tissue damage across a range of pathological conditions (40).

Primary infection with *E. caproni* also impacted several processes related to epithelial homeostasis and immunity, which may be critical for understanding both the susceptibility to infection and the development of chronic infections in mice (41). Notably, murine susceptibility was associated with tissue hyperplasia resulting from impaired epithelial renewal induced by the parasite. This was characterized by high levels of crypt-cell proliferation and a reduced rate of epithelial turnover, ultimately disrupting tissue homeostasis. In contrast, the preservation of epithelial homeostasis has been linked to resistance against *E. caproni* infection (19, 20). Strikingly, our GSEA-KEGG analysis showed a marked reduction in the expression of tight junction and GAP junction proteins, structures essential for the maintenance of epithelial integrity and architecture (42). Among the downregulated proteins were isoforms of cytoplasmic tubulins and actins, whose importance in sustaining enterocyte tight junction complexes is well established (43). The disruption of these structural components compromises intestinal epithelial integrity and is associated with increased permeability, facilitating the translocation of microbial products and luminal antigens. This, in turn, can lead to immune cell activation and the onset of exacerbated inflammatory responses (44). Indeed, in murine models of experimental colitis, β-actin deficiency in the cytoplasm has been linked to an altered epithelial phenotype marked by increased intestinal permeability (45). Furthermore, the present findings suggest that primary *E. caproni* infection may interfere with key mechanisms that enable effective remodeling of the intestinal epithelium. Supporting this, both ORA and GSEA analyses revealed functional enrichment in the underexpression of calcium-binding and nucleotide-binding proteins involved in epithelial turnover processes such as endocytosis (46, 47). Interestingly, this process has also been implicated in the regulation of tight junction dynamics (48).

Among the underexpressed proteins, a substantial number of GTPase subunits, including Ras-related (Rab) proteins, ADP-ribosylation factors (ARF), and annexin isoforms, were identified, all of which are critically involved in vesicular trafficking and tissue remodeling processes (46, 47, 49–51). For instance, several studies have demonstrated that Rab8, Rab11a, and ARF proteins are essential for the proper transport of E-cadherin (52–54). Furthermore, experimental evidence indicates that occludin-deficient mice exhibit altered histological phenotypes, characterized by tissue hyperplasia, chronic inflammation, and impaired epithelial barrier functions (55), a pattern closely resembling the histopathological features associated with susceptibility to primary *E. caproni* infection in mice (56). These findings are particularly relevant in the context of intestinal helminthiases, where proteases derived from the excretory/secretory products of *Trichinella spiralis* have been shown to degrade structural proteins essential for maintaining intestinal epithelial integrity (57).

Annexins also play a key role in stabilizing tight junctions through their interaction with the actin cytoskeleton (58, 59). Therefore, the downregulation of these proteins and the consequent suppression of their signaling pathways—together with impairment of the mechanisms supporting efficient tissue repair—appear to be critical in determining susceptibility to primary *E. caproni* infection. This occurs within an environment characterized by increased intestinal permeability and limited IL-25 production (41, 60, 61).

Our ORA analysis revealed functional dysregulation of processes mediated by antimicrobial proteins and peptides (AMPs), characterized by increased expression of defensins, regenerative proteins, galectins, and interferon-stimulated gene 15 (ISG15), accompanied by a marked downregulation of proteins derived from eosinophils, neutrophils, gasdermins, intelectins, and angiogenins. In the intestine, AMPs not only serve as antimicrobial agents but also play a crucial role in maintaining intestinal homeostasis, as they are involved in the regulation of inflammation and the composition of the microbiota (62). Furthermore, since AMPs are concentrated near the epithelial surface, they reinforce the mucosal barrier, thereby preventing the penetration of pathogens (63, 64).

Among the upregulated proteins, α-defensins (cryptdins in rodents), specifically Defa4 and Defa-rs2, are abundant in the ileum and help restrict local colonization by Gram-positive and Gram-negative bacteria. Their expression is not limited to bacterial stimuli, as they also respond to fungi, viruses, parasites, and inflammation from gastrointestinal damage (65, 66). Helminth infections (e.g., *Nippostrongylus brasiliensis, T. spiralis*) increase α-defensin expression of intestinal Paneth cells (67, 68). These peptides are stored as inactive pro-α-defensins and activated by MMP7 (69). Upon stimulation of Toll-like and NOD-like receptors, they are secreted into the intestinal lumen along with pro-inflammatory cytokines and antimicrobial peptides (70, 71). Paneth cell degranulation is primarily triggered by IFN-γ, not directly by bacteria, and also induces mucus secretion from goblet cells (72). α-defensins contribute to gut microbiota homeostasis, with cryptdin 4 (Defa4) showing the highest antimicrobial activity during dysbiosis (73). In *E. caproni*-infected mice, Regenerating Islet-Derived (Reg) proteins were also overexpressed. These proteins have bactericidal, pro-proliferative, differentiation-promoting, and anti-apoptotic roles during tissue damage or inflammation (74, 75). Reg1 supports intestinal cell growth and villus structure, contributing to the maintenance of consistent villus architecture and length (76), and reduces epithelial apoptosis during inflammation (77). Both Reg3b and Reg3g are upregulated during infection and also regulate the gut microbiota (78). In Reg3g knockout mice, altered mucosal layer distribution—especially at villus tips—led to increased contact between microbiota and ileal epithelium, favoring bacterial invasion (79).

Although AMPs are generally protective, their overexpression—particularly during chronic inflammation or persistent infection—may have pathological consequences, such as increased permeability and dysbiosis (80, 81), an effect previously observed in the context of *E. caproni* infections (82). Thus, defining the role of AMPs in parasitic infections remains a challenge, as their immunomodulatory functions are well documented (83). In this context, the observed changes in the expression of these proteins may be significant for understanding susceptibility to *E. caproni* infection, particularly in relation to the development of Th1-type immune responses and a lack of responsiveness to IL-25. Several studies have reported that high expression of intelectin-1b is associated with the induction of Th2 responses via the regulation of IL-25, IL-33, and thymic stromal lymphopoietin (TLSP) (84). Likewise, treatment with recombinant IL-25 has been shown to upregulate defensins, angiogenins, and mast cell metallopeptidases (85, 86). Similarly, research on experimental nematode infections has demonstrated that the combined action of Th2 responses and AMPs (including angiogenins, resistins, and intelectins) contributes to the effective expulsion of intestinal helminths (87–89). In line with these findings, our study revealed a downregulation of Ang-4, which may reflect a reduced capacity to control the establishment of a helminth infection. A Th1-biased immune profile, as typically observed during primary infection with *E. caproni*, does not favor Ang4 induction, thereby compromising host defense mechanisms against helminths, which generally require a robust Th2 response for effective elimination (86, 89).

We also observed overactivation of NOD-like receptor-dependent signaling pathways, as revealed by GSEA-KEGG analysis. Some of these receptors are notable for their specific activation upon recognition of bacterial cell wall components (90). Their activation has been linked to the modulation of mitogen-activated protein kinase (MAPK), nuclear factor kappa B (NF-κB), and type I interferon signaling pathways (91). However, our results also indicate negative regulation of the MAPK pathway, suggesting that the pronounced inflammatory response induced by primary *E. caproni* infection in mice is largely independent of the MAPK pathway. In this context, the marked overexpression of several interferon-stimulated genes (ISGs), such as ISG15 (+5.51), appears particularly relevant. The nuclear translocation of interferon regulatory factors (IRF), such as IRF3 and IRF7, stimulated by NOD and NF-κB, promotes the expression of type I interferons. These, in turn, mediate signal transduction through the Janus Kinase (JAK/STAT) pathway in order to promote gene expression of ISGs (92–94). ISG15 has been shown to enhance the production of IFNγ, a cytokine closely associated with susceptibility to primary *E. caproni* infection (95–98). Thus, these facts can be indicative of the activation of NOD receptors, and their subsequent implications could be factors that determine the development of an adaptive immune response, such as the Th1 phenotype, which has been observed in high-compatibility hosts (41).

The overexpression of pathways associated with N-glycan biosynthesis suggests an increase in protein glycosylation following primary *E. caproni* infection. This finding aligns with previous reports indicating that such infections induce substantial alterations in the composition and structure of intestinal mucus in ICR mice, correlating with susceptibility (99). Finally, among the most notable biological processes, a functional enrichment of the lymphocyte-mediated immune response was found. Among the proteins implicated were galectin-9 (Gal-9), β2-microglobulin, carcinoembryonic antigen-related cell adhesion molecule 1, and the membrane-bound form of dipeptidyl peptidase 4 (DPP4). In chronic *Toxoplasma gondii* infections in BALB/c mice, a positive correlation has been observed between the expression of Gal3 and Gal9 with INFγ and ArgI, supporting the role for lectins in Th1 cell polarization (100). In addition to their immunomodulatory function, Gal-9 also contribute to intestinal epithelial repair by regulating cell proliferation and mucosal restitution following tissue injury (101). Gal-9 also mediates eosinophil adhesion to the surface of fibroblasts and endothelial cells. However, Gal-9 is also important in acquired immunity because it can induce dendritic cell maturation and IL-12 secretion, leading to a Th1 response (102). Similarly, DDP4 has been shown to promote Th1 polarization, correlating with increased production of Th1-associated cytokines (103).

**Figure 10:**
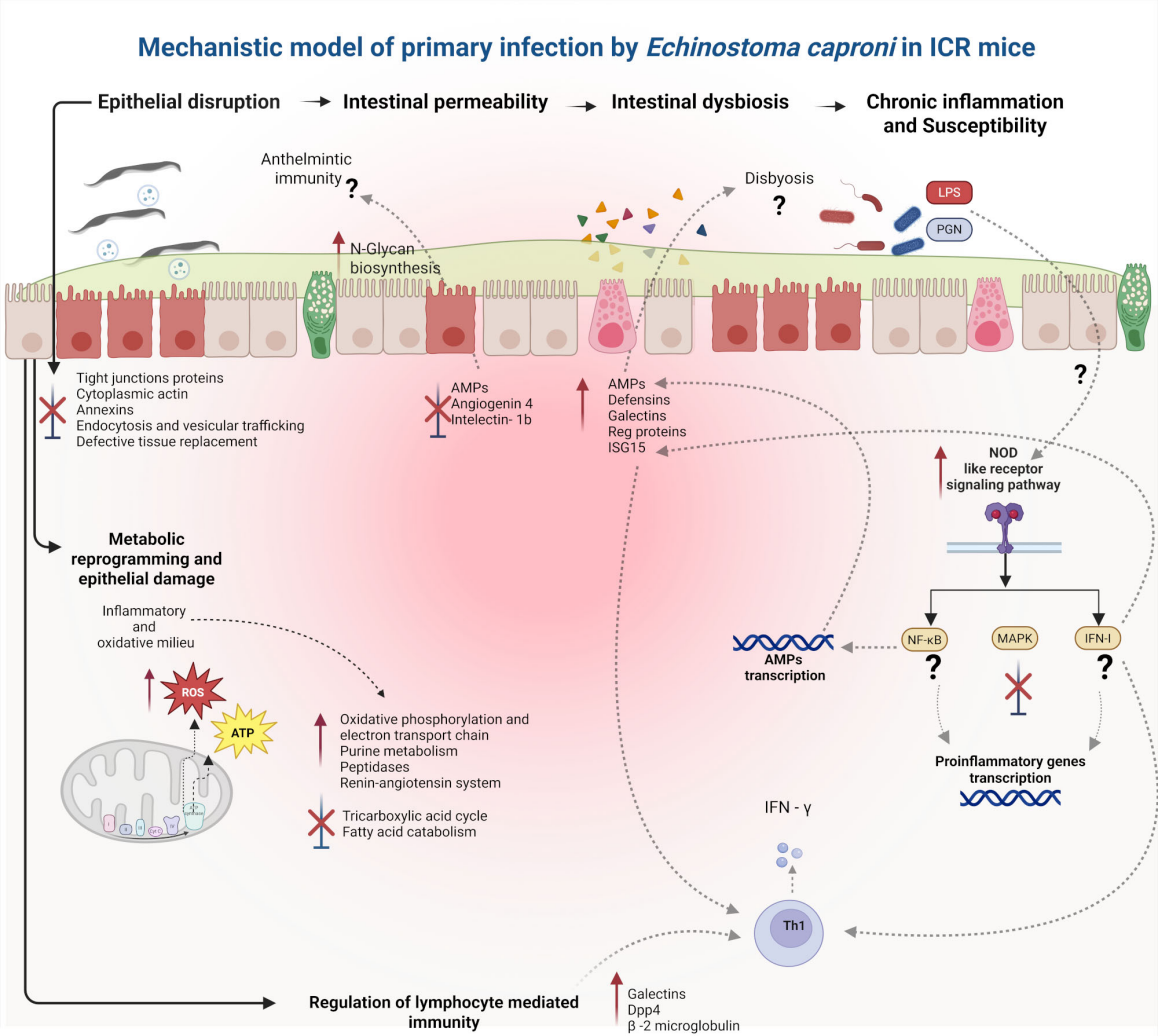
Mechanistic model of omics-driven alterations in the small-intestinal epithelium of ICR mice during primary *Echinostoma caproni* infection. Primary *E. caproni* infection induces a profound disruption of intestinal homeostasis, characterized by epithelial barrier breakdown and impaired epithelial turnover. These alterations are associated with the loss of structural and tight-junction proteins, defective vesicular trafficking, and compromised tissue repair, potentially leading to increased intestinal permeability and dysbiosis. In response, the host exhibits excessive mucus production and an imbalanced activation of antimicrobial peptides (AMPs), including defensins, galectins, regenerative proteins, and ISG15, alongside suppression of other epithelial antimicrobial effectors. Concurrently, the presence of worms in the intestinal lumen drives epithelial metabolic reprogramming, marked by mitochondrial dysfunction, altered oxidative phosphorylation and purine metabolism, reduced ATP production, and increased oxidative stress. These changes coincide with activation of innate immune recognition pathways, particularly NOD-like receptor signaling, converging on pro-inflammatory pathways and potentially promoting Th1 immune responses. Collectively, the integration of epithelial disruption, metabolic dysfunction, and dysregulated immune activation could be the cause of the establishment of chronic inflammation and increased host susceptibility during primary *E. caproni* infection.

In summary, our findings support the hypothesis that susceptibility to primary *E. caproni* infection is associated with the establishment of a proinflammatory milieu, driven by infection-induced metabolic alterations that promote cellular damage through the accumulation of reactive oxygen species and lipids. This is accompanied by a progressive loss of epithelial integrity and homeostasis, evidenced by the downregulation of proteins essential for epithelial barrier remodeling and stability. The resulting tissue imbalance is characterized by increased intestinal permeability and enhanced antigen recognition, leading to conformational changes in the chemical barriers of the intestinal epithelium mediated by dysregulated production of AMPs, altered mucus composition, and activation of immune cells (**Figure 10**).

Understanding the biological meaning of the proteomic changes observed in this study requires consideration of several, not mutually exclusive, interpretations. First, some of the differentially expressed proteins may reflect host defense mechanisms, representing an attempt to limit parasite establishment, modulate inflammation, or restore tissue homeostasis. Conversely, certain alterations could result from parasite-driven manipulation, in which the pathogen actively modulates host signaling pathways to enhance its survival, immune evasion, or nutrient acquisition. A third possibility is that the observed patterns represent pathological consequences of infection, arising from tissue damage, metabolic stress, or dysregulated immune responses. Importantly, these explanations are not mutually exclusive, and the proteomic signature we report is likely the product of a complex interplay between host responses, parasite-mediated modulation, and infection-induced pathology. Future mechanistic studies will be required to disentangle the relative contribution of each of these processes. Collectively, these findings contribute novel insights into the complex host-parasite interactions that underlie susceptibility to intestinal helminth infections and may inform the development of future therapeutic or preventive strategies.

## Data Availability

The proteomic data have been deposited to the ProteomeXchange Consortium via the PRIDE partner repository with the Project accession: PXD068954 (The full list of data can be found in the **Supplementary Files**).
