# Supplementary material for: Susceptibility of mice to primary Echinostoma caproni infections is associated with metabolic and structural changes
Source: Front Immunol. 2026 Apr 21;17:1720183. doi: 10.3389/fimmu.2026.1720183 (PMC13139024; doi:10.3389/fimmu.2026.1720183)
Supplement: Supplementary file 4 [file DataSheet1.pdf]

```
#  C  = Control
#  I  = Infected




library(glmnet)
library(car)
library(caret)
library(openxlsx)
library(NMF)




#
# Caso 2: Control vs Infected
#
###########################################


############################

datos<-read.csv2("datos.csv",  stringsAsFactors=FALSE, fileEncoding="latin1")
names(datos)
attach(datos)


Protein<-as.vector(datos[,1])

datos1<-data.frame(apply(datos[,-1],2,log2))

names(datos1)

#############################################




#Lasso:
library(glmnet)
library(caret)
datos3<-data.frame(t(datos1[,1:8]))


#Lasso binomial :
################################
##Comparacions:

#1) Control vs Infected

tipotrat <- factor(c(rep(0,4),rep(1,4)),levels=0:1,labels=c("Control","Infected"))

normalizar<-t(scale(t(datos1[,1:8])))


annotation = data.frame(type = tipotrat)
pdf(file="../LATEX/Figures/ControlvsInfected/heatmap_normalizado.pdf",width =8, height = 6,onefile=
FALSE)
aheatmap(normalizar, scale="row", color=c("green", "darkgreen","black","darkred","red"),annCol =
annotation,labRow = NA,fontsize = 7,cexRow = 0.7,annColors='Set1')
```

```
dev.off()
set.seed(123)
fit_lasso<-glmnet(as.matrix(datos3), tipotrat, nlambda=100, alpha=1, family="binomial")
plot(fit_lasso, label=TRUE)

cv <- cv.glmnet(as.matrix(datos3), tipotrat, alpha=1, nfolds=12, family="binomial")
plot(cv)
#Valor lambda con el que se consigue el mínimo test-error
l1<-cv$lambda.min  #0.00494543

lambdas.min<-replicate(100, cv.glmnet(as.matrix(datos3), tipotrat, alpha=1, nfolds=10,
family="binomial")$lambda.min)

plot(density(lambdas.min))

median(lambdas.min)


l <- median(lambdas.min)   #0.00494543
l3<-cv$lambda.1se #  0.2701331
#Coeficientes del modelo
res <- as.matrix(predict(fit_lasso, newx=as.matrix(datos3),s=l, type="coefficients"))
res1 <- as.matrix(predict(fit_lasso, newx=as.matrix(datos3),s=l3, type="coefficients"))


coeficientes_lasso<-res[res!=0][-1]
coeficientes_lasso1<-res1[res1!=0][-1]

#coeficientes_lasso2<-res2[res2!=0][-1]


#Filas seleccionadas(haciendo [-1] quitamos la interceptación
proteinas_lasso<-(which(abs(res)>0)-1)[-1]
# 34  364  487  738 1470 1650

proteinas_lasso_1se<-(which(abs(res1)>0)-1)[-1]
# 364  487  738 1650

nom_proteinas_lasso<-Protein[proteinas_lasso]
Resultados_T1vsT2<-
data.frame(Fila=proteinas_lasso,Proteína=nom_proteinas_lasso,Coeficiente=coeficientes_lasso)

write.xlsx(Resultados_T1vsT2, "../Resultados/ControlvsInfected/Proteinas_lasso.xls",overwrite = TRUE)


#proteins identified by Lasso

pdf(file="../LATEX/Figures/ControlvsInfected/heatmap_lasso.pdf",width = 6, height = 6,onefile= FALSE)

aheatmap(t(scale(datos3[,proteinas_lasso])), scale="row", color=c("green",
"darkgreen","black","darkred","red"),annCol = annotation,labRow=nom_proteinas_lasso,fontsize =
7,cexRow = 0.7,annColors='Set1')

dev.off()

####################################
#Elastic Net
###############################


fit_net_bin<-glmnet(as.matrix(datos3), tipotrat, nlambda=100, alpha=1, family="binomial")
```

```
prueba<-glmnet(x = as.matrix(datos3), y=tipotrat,nlambda=100, alpha=0, family="binomial")   #
[4.94,494.5]
prueba<-glmnet(x = as.matrix(datos3), y=tipotrat, nlambda=100, alpha=1, family="binomial")   #
[0.00494, 0.4945]
#Para la secuencia de lambda se espacian más los valores de elevados de lambda
red <- expand.grid(.alpha = seq(0.1,1, 0.1), .lambda = exp(seq(log(0.00494),log(494), length=200)))

Control <- trainControl(verboseIter =TRUE,method="cv")
netFit <- train(x = as.matrix(datos3), y=tipotrat,method = "glmnet", tuneGrid = red, trControl = Control,
family="binomial")

## alpha = 0.1, lambda = 0.803
plot(netFit)
attributes(netFit)
netFit$bestTune



a<-netFit$bestTune[,1]
l<-netFit$bestTune[,2]


datos5<-datos3

#alpha=a
fit<-glmnet(as.matrix(datos5), tipotrat,alpha=a, family="binomial")  #alpha 1 = Lasso, alpha 0=Ridge
plot(fit, label=TRUE)


res <- as.matrix(predict(fit, newx=as.matrix(datos5),s=l, type="coefficients"))



coeficientes_elasticNet<-res[res!=0][-1]

proteinas_elasticNet<-(which(abs(res)>0)-1)[-1]
nombre_proteina_elasticNet<-Protein[proteinas_elasticNet]
#Proteins alpha = 0.2, lambda = 0.04:

#4    6    9   10   23   24   25   30   32   34   35   40   42   59   62   63   67   73   87   88   89   92   94
#   99  101  102  103  106  108  110  114  117  126  127  129  132  150  153  158  161  162  169  170
183  185  186
#  188  192  212  223  234  237  252  270  276  283  286  291  294  297  328  329  336  338  357  358
359  360  364
#  379  382  393  395  396  400  403  408  430  447  454  456  463  468  471  473  479  480  486  487
490  492  508
#  510  512  529  539  547  555  560  565  574  581  586  588  591  594  597  618  620  628  635  636
638  639  640
#  643  666  668  681  683  686  689  690  693  702  722  727  730  738  754  764  773  777  780  784
785  787  792
#  795  797  800  842  848  857  859  864  866  867  869  874  881  882  887  898  900  917  919  925
934  937  942
#  946  949  964  971  973 1017 1024 1039 1048 1074 1075 1082 1101 1105 1107 1108 1117 1139
1140 1143 1159 1168 1174
# 1177 1188 1200 1201 1215 1231 1236 1244 1261 1270 1277 1289 1291 1293 1297 1310 1318 1320
1344 1369 1384 1386 1390
# 1394 1417 1430 1433 1438 1446 1459 1464 1470 1477 1499 1504 1511 1565 1567 1597 1619 1628
1642 1643 1650 1657 1689
# 1693 1696 1705 1721 1754 1773 1795

#All proteins by Lasso are with elasticNet
```

```
Resultados_elasticnet<-
data.frame(Variable=proteinas_elasticNet,Proteína=nombre_proteina_elasticNet,Coef=coeficientes_elastic
Net)

write.xlsx(Resultados_elasticnet, "../Resultados/ControlvsInfected/Resultados_elasticnet.xls",overwrite =
TRUE)



pdf(file="../LATEX/Figures/ControlvsInfected/heatmap_elasticnet.pdf",width = 8, height = 6,onefile=
FALSE)
aheatmap(t(scale(datos3[,proteinas_elasticNet])), scale="row", color=c("green",
"darkgreen","black","darkred","red"),annCol = annotation,fontsize = 7,cexRow =
0.7,labRow=nombre_proteina_elasticNet,annColors='Set1')
dev.off()


#################################################
#
#plsda with mixOmics
#
#################################################
library(mixOmics)

datos3<-data.frame(t(datos1[,1:8]))

#PCA (normal)
pca<- pca(datos3, ncomp = 3, scale = TRUE, center = TRUE)



pdf(file="../LATEX/Figures/ControlvsInfected/pca.pdf",width = 8, height = 6,onefile= FALSE)
plotIndiv(pca, group = tipotrat,col.per.group=c("red","blue"),
        title = 'PCA , comp 1 - 2',legend = TRUE)

dev.off()



#PLS-DA  :
###################

#PLS-DA classic
plsda <- plsda(datos3, tipotrat, ncomp = 3)




pdf(file="../LATEX/Figures/ControlvsInfected/plsda.pdf",width = 8, height = 6,onefile= FALSE)
plotIndiv(plsda,
        group = tipotrat, ellipse = TRUE, col.per.group=c("red","blue"),
        legend = TRUE,
        title = 'Classical PLS-DA')

dev.off()


#R²X: La primera componente explica un 39.8% de la varianza de las variables. La 2ª componente
explica un 16.3% de la varianza de las variables que nos queda por explicar.

plsda$prop_expl_var$X
```

```
# R²Y:La primera componente explica toda la varianza de la respuesta Y. La segunda componente es la varianza residual.Esto significa que la separación de los grupos ya se determina prácticamente toda con la primera componente y la segunda sólo aporta información marginal.

plsda$prop_expl_var$Y

##Validez y capacidad predictiva
perf.plsda <- perf(plsda,
                   validation = "loo",
                   progressBar = TRUE)
str(perf.plsda)

#Q² values:  En este tenemos que 1 de 8 muestras se clasifica mal, luego el modelo en este caso no es malo pero para un n=4 no es un resultado robusto.
perf.plsda$error.rate$overall
#         max.dist   centroids.dist    mahalanobis.dist
#comp1    0.125          0.125             0.125
#comp2    0.125          0.125             0.125



#####################################################################
######################################################
#function VIP-score

resultado.vip <- vip(plsda)
head(resultado.vip)
#nos quedamos sólo con los resultados que muestra cuando cogemos tres componentes y
#las ordenamos
vip.comp2<- resultado.vip[,1]
vip.ordenadas2 <- sort(vip.comp2)
#???Proteínas con un vip mayor a 1
proteinas_mayor1<-unique(names(which(vip.ordenadas2>=1)))
proteinas_mayor1<-as.factor(proteinas_mayor1)
proteinas_mayor1_num<-as.numeric(substr(proteinas_mayor1,2,5))
proteinas_plsdamayor1<-Protein[proteinas_mayor1_num]
proteinas_vip1<-data.frame(proteinas_mayor1_num,proteinas_plsdamayor1)
colnames(proteinas_vip1)<-c("NumProteina","Proteina")
write.xlsx(proteinas_vip1, "../Resultados/ControlvsInfected/Resultados_vip_mayor1.xls",sheetName = "Proteinas")


#Para un VIP>1.5 no tenemos ninguna proteina
proteinas_mayor15<-unique(names(which(vip.ordenadas2>=1.5)))
proteinas_mayor15<-as.factor(proteinas_mayor15)
proteinas_mayor15_num<-as.numeric(substr(proteinas_mayor15,2,5))
sort(proteinas_mayor15_num)
proteinas_plsdamayor15<-Protein[proteinas_mayor15_num]
resultados_vipmayor15<-data.frame(Fila=proteinas_mayor15_num,Proteínas=proteinas_plsdamayor15)

write.xlsx(resultados_vipmayor15,
"../Resultados/ControlvsInfected/Resultados_vip_mayor15.xls",sheetName = "Proteinas vip15",overwrite =TRUE)

#####################################################################
#################
#### heatmap only proteins vip>15 ###########
#####################################################################
#################
#
```

```
pdf(file="../LATEX/Figures/ControlvsInfected/heatmap_vip1.pdf",width = 8, height = 6,onefile= FALSE)

aheatmap(t(scale(datos3[,proteinas_mayor1_num])), scale="row", color=c("green",
"darkgreen","black","darkred","red"),annCol = annotation,labRow=proteinas_plsdamayor1,fontsize =
7,cexRow = 0.7,annColors='Set1')
dev.off()

pdf(file="../LATEX/Figures/ControlvsInfected/heatmap_vip15.pdf",width = 8, height = 6,onefile= FALSE)

aheatmap(t(scale(datos3[,proteinas_mayor15_num])), scale="row", color=c("green",
"darkgreen","black","darkred","red"),annCol = annotation,labRow=proteinas_plsdamayor15,fontsize =
7,cexRow = 0.7,annColors='Set1')
dev.off()

###############   ANALISIS with  LIMMA   ################################



library("limma")




#Proteinas que se transfieren a distintas temperaturas:

design <- model.matrix(~ 0+tipotrat)
colnames(design) <- c("Control","Infected")


fit <- lmFit(datos1[,1:8], design)
fit <- eBayes(fit)
#Coef especifica el contraste del modelo lineal de interés
topTable(fit,coef=1,number=1809,genelist=Protein,adjust.method="BH")
topTable(fit,coef=2,number=1809,genelist=Protein,adjust.method="BH")
#En la siguiente salida lo que hace es como un anova, ve las diferencias de los 4 tiempos(niveles) por
eso saca el test F(en
#las salidas anteriores muestras el test t pq lo hace dos a dos. Las proteínas seleccionadas son las misma
y los fold-change son
#los mismos. El valor AveExpr es la media de todos los fold-changes de los diferentes tiempos).

toptable_todas<-topTable(fit,number=1809,genelist=Protein,adjust.method="BH")
write.xlsx(toptable_todas, "../Resultados/ControlvsInfected/toptable_individuales.xls",overwrite =TRUE)

#Vamos a ver las proteínas haciendo todas las posibles comparaciones:
#contrast.matrix <- makeContrasts(H0-L0, levels=design)
contrast.matrix <- makeContrasts(Control-Infected, levels=design)
fit3 <- contrasts.fit(fit, contrast.matrix)
fit3 <- eBayes(fit3)

topTable(fit3,coef=1,number=1809,genelist=Protein,adjust.method="BH")


top_comparaciones<-topTable(fit3,number=1809,genelist=Protein,adjust.method="BH")
write.xlsx(top_comparaciones, "../Resultados/ControlvsInfected/top_comparaciones.xls",overwrite
=TRUE)

results3 <- decideTests(fit3,method="global")
data_results3<-summary(results3)

tabla_diamVenn_comparaciones<-data.frame(Protein,results3)
write.xlsx(tabla_diamVenn_comparaciones,
```

```
"../Resultados/ControlvsInfected/tabla_diamVenn_comparaciones.xls",overwrite =TRUE)

write.xlsx(data_results3, "../Resultados/ControlvsInfected/tabla_diagrama_Venn.xls",overwrite =TRUE)


pdf(file="../LATEX/Figures/ControlvsInfected/volcano_diferencias.pdf",width =8, height = 6,onefile=
FALSE)
par(mfrow=c(2,1))
volcanoplot(fit3,coef=1,highlight=10,names=Protein,main="ControlvsInfected",cex=0.6)
vennDiagram(results3, cex=0.8,
            include=c("up", "down"),
            counts.col=c("red", "blue"),
            circle.col = c("red", "blue", "green3"))
dev.off()

par(mfrow=c(1,1))
```
